# Unlocking the Microbiome Communities of Banana (*Musa* spp.) under Disease Stressed (*Fusarium* wilt) and Non-Stressed Conditions

**DOI:** 10.3390/microorganisms8030443

**Published:** 2020-03-20

**Authors:** Manoj Kaushal, Rony Swennen, George Mahuku

**Affiliations:** 1 International Institute of Tropical Agriculture (IITA), Mikocheni B, Dar es Salaam 34441, Tanzania; g.mahuku@cgiar.org; 2 International Institute of Tropical Agriculture (IITA), Arusha 447, Tanzania; rony.swennen@kuleuven.be; 3 Laboratory of Tropical Crop Improvement, Division of Crop Biotechnics, KU Leuven, B-3001 Leuven, Belgium; 4 Bioversity International, Willem De Croylaan 42, B-3001 Leuven, Belgium

**Keywords:** *Musa*, *Fusarium* wilt, rhizosphere, microbiome diversity, *Fusarium oxysporum* f. sp. *cubense*

## Abstract

We assessed the diversity, structure, and assemblage of bacterial and fungal communities associated with banana plants with and without *Fusarium oxysporum* f. sp. *cubense* (Foc) symptoms. A total of 117,814 bacterial and 17,317 fungal operational taxonomy units (OTUs) were identified in the rhizosphere, roots, and corm of the host plant. Results revealed that bacterial and fungal microbiota present in roots and corm primarily emanated from the rhizosphere. The composition of bacterial communities in the rhizosphere, roots, and corm were different, with more diversity observed in the rhizosphere and less in the corm. However, distinct sample types i.e., without (asymptomatic) and with (symptomatic) *Fusarium* symptoms were the major drivers of the fungal community composition. Considering the high relative abundance among samples, we identified core microbiomes with bacterial and fungal OTUs classified into 20 families and colonizing distinct plant components of banana. Our core microbiome assigned 129 bacterial and 37 fungal genera to known taxa.

## 1. Introduction

Rhizospheric and endophytic microbiota have been shown to significantly improve plant health and development [1,2]. Beneficial microorganisms in the rhizosphere/roots of bananas have been investigated for potential beneficial impacts [3]. Both roots as well as the thin soil layer adhering to roots, called rhizosphere, host diverse microbial communities. To date, however, most research has focused on only a small fraction of a pre-targeted group of the rhizosphere microbiota. Hence information is missing of the entire associated microbial diversity and influencing factors.

Bananas (*Musaceae* spp.) are cultivated throughout the humid tropics and sub-tropics. In sub-Saharan Africa (SSA), bananas are an important component of the diet and a crucial source of income for many small holder farmers. Disease pressure is the major threat threatening banana productivity in the region [4,5,6]. The devastation of bananas by Panama disease caused by the soil borne *Fusarium oxysporum* f. sp. *cubense* (Foc), lack of pesticides, and appropriate resistant varieties encouraged the search for alternatives to sustain productivity. Foc race 1 is present everywhere in SSA hampering dessert banana (of the *Musa* subgroup AAB) productivity [7]. In addition, Foc race 4 is present in Mozambique [3]. The pathogen can spread from infected to non-infected fields through banana suckers, water, and movement of people. Foc enters through the roots and infects the plant vascular system. Initial disease expression is yellowing of leaves. In later stages, maroon color lines appear inside the pseudostem. To date, no effective control exists against this devastating disease, and the pathogen can persist in the soil for decades without a suitable host. In this context, and since Foc is soil borne, the study of host-microbiome interactions and defense against phytopathogens might open new ways to improve banana growth and yield [8,9]. Endophytes isolated from banana suckers exhibit a significant antagonistic activity against phytopathogens and should be explored as self-supporting microbial ecosystems [10]. The advent of high-throughput sequencing technologies allows the systematic study of plant-associated microbial communities [11,12]. However, there are gaps to be resolved between plant–microbe interactions and their underlying mechanisms [13,14] in support of enhanced production.

To elucidate the role of the microbiome in plant growth and disease control, it is necessary to investigate the microbiome dynamics and distribution in different plant components. Thus, the present investigation was conducted to study the microbiome (both bacteria and fungi) structure and mode of associations with asymptomatic and symptomatic banana plants infected by *Fusarium oxysporum* f. sp. *cubense* (Foc) race 1. Symptomatic banana plants were selected based on the morphological symptoms including yellow coloration of leaves and maroon color lines inside the pseudostem. In addition, we demonstrated the composition and assemblage of the naturally occurring microbiome in the rhizosphere, root, and corm samples. Our study is the first to describe an inventory of bacterial and fungal communities associated with the components of asymptomatic and symptomatic banana plants infected by Foc. In addition, we describe core microbiomes in the rhizosphere, root, and corm and established a model for studying banana microbiota.

## 2. Materials and Methods

### 2.1. Sample Collection and DNA Extraction

**Figure 1:**
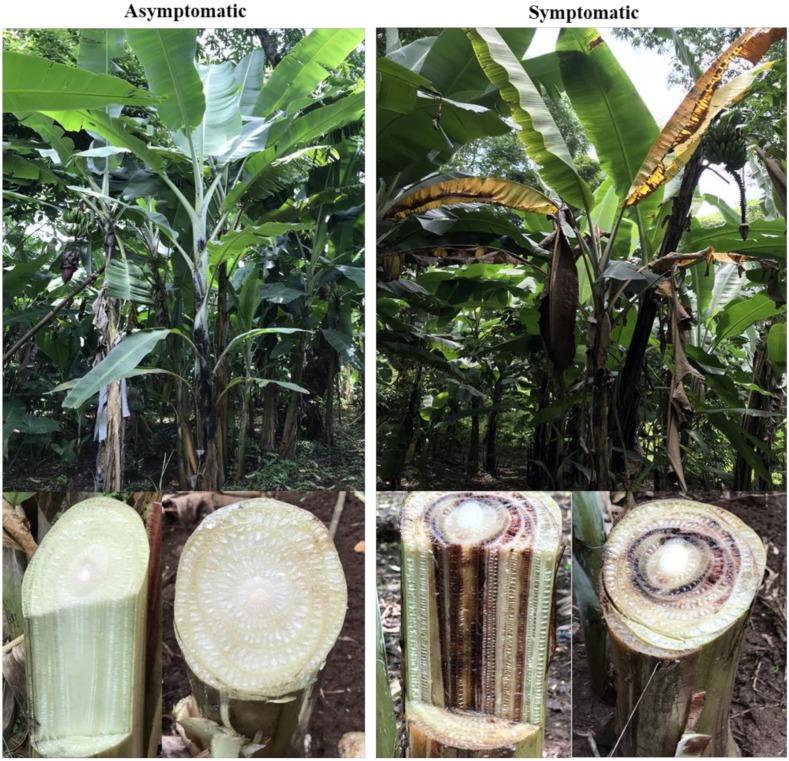
Banana plants under non-stressed (asymptomatic) and disease stressed conditions (symptomatic) caused by *Fusarium oxysporum*.

Sukari Ndizi (*Musa* subgroup AAB) is a popular dessert banana cultivar in east Africa that is susceptible to Foc. The rhizosphere, roots, and corm of asymptomatic and symptomatic plants were targeted for sampling. Symptomatic banana plants exhibited yellowing leaves with brown streak discoloration inside the pseudostem and corm, and asymptomatic plants had green leaves with no discoloration inside the pseudostem and corm (Figure 1). Samples were collected from three random locations around a single plant at a distance of 15 cm. Rhizosphere samples were composed of soil attached to roots at a depth of 15–30 cm. Root samples were collected at the same depth after the removal of attached soil. Corm samples were collected from a depth of 15–50 cm and included the outer 2–3 cm of the cortex. Each composite sample was made from five different plants from four independent fields (a total of 20 plants). A total of 12 composite samples were collected from two locations in Tanzania, i.e., Arusha (3°22′29.6″S, 36°48′16.8″E) and Kilimanjaro (3°14′14.6″S, 37°15′03.7″E) (Figure S1). Samples were put in polythene bags and kept in an ice-cold box until transported to the laboratory. In the laboratory samples were further kept at 4 °C until processed. DNA was extracted using the Plant and Soil DNA Isolation Kit (Zymo Research Corp., Irvine, CA, USA).

### 2.2. Library Preparation

The V3–V4 hyper-variable region of the 16S rDNA gene of bacteria and archaea and internal transcribed spacer (ITS2) of fungi was amplified (Table S2). Amplification was done with i5 and i7 primers as per the standard Illumina protocol [15]. The amplicon library was prepared by Nextera XT Index Kit (Illumina Inc.) as per the 16S Metagenomic Sequencing Library preparation protocol. The amplicon libraries were purified by 1X AMpureXP beads, checked on Agilent DNA1000 chip on Bioanalyzer2100 and quantified by Qubit Fluorometer 2.0 using Qubit dsDNA HS Assay kit (Life Technologies).

### 2.3. Cluster Generation and Sequencing

The library was loaded onto the Illumina platform at a concentration of 10–20 pM for cluster generation and sequencing. The template fragments were sequenced in both the forward and reverse direction through Paired-End sequencing on the Illumina platform.

### 2.4. Data Processing and OTU Clustering of 16S and ITS2 Amplicons

DNA (chimeras) sequences were filtered using usearch61 algorithm (de novo mode) and generated in silico using the FLASH tool with a minimum 10 bp overlap to recreate the V3–V4 region. All the samples were pooled in a single file, processed, and clustered to facilitate resemblance among different plant components and locations. The taxonomic names were assigned with UCLUST algorithm. OTUs for bacterial and fungal communities correlated with their abundances, were organized into a BIOM file. QIIME was used for downstream processing and intra-sample analysis or Alpha (α) and Beta (β) diversity calculations to analyze species richness. We used non-metric multidimensional scaling (NMDS) and principal coordinate analysis (PCoA) plots to visualize differences in bacterial and fungal communities composition among samples.

### 2.5. Diversity Metrics

Differences in microbial community richness among rhizosphere, root, and corm of banana were evaluated with chaos1 estimator and the Shannon diversity index. The Bray–Curtis dissimilarity matrix was calculated for bacterial and fungal communities and used for PCoA analysis with QIIME.

### 2.6. Characterizing OTU Core and Non-Core Communities

We investigated core and non-core microbial communities (both for bacteria and fungi) in combination of sample type and location with MySQL query over the filtered OTU table. Further, we evaluated the relative abundance of each OTU in different samples and taxonomical histograms were plotted.

### 2.7. Nucleotide Sequence Accession Numbers

The sequence data were deposited in NCBI with SRA accession PRJNA493905 and PRJNA494050.

## 3. Results

### 3.1. General Characteristics of Banana Microbiome

We characterized the bacterial and fungal communities associated with each plant component (rhizosphere, root, and corm), location, and sample types (asymptomatic and symptomatic). A total of 16 million reads, with an average of 0.9 and 0.5 million reads per sample type, were generated for 16S and ITS2, respectively. A total of 4.6 million reads from 16S and 2.5 million reads from ITS2 were clustered at ≥97% sequence identity after the removal of low-quality reads via the UCLUST algorithm. A total of 117,814 bacterial and 17,317 fungal operational taxonomy units (OTU) were recovered.

### 3.2. Banana Rhizosphere: A Rich Microbiome Reservoir

**Figure 2:**
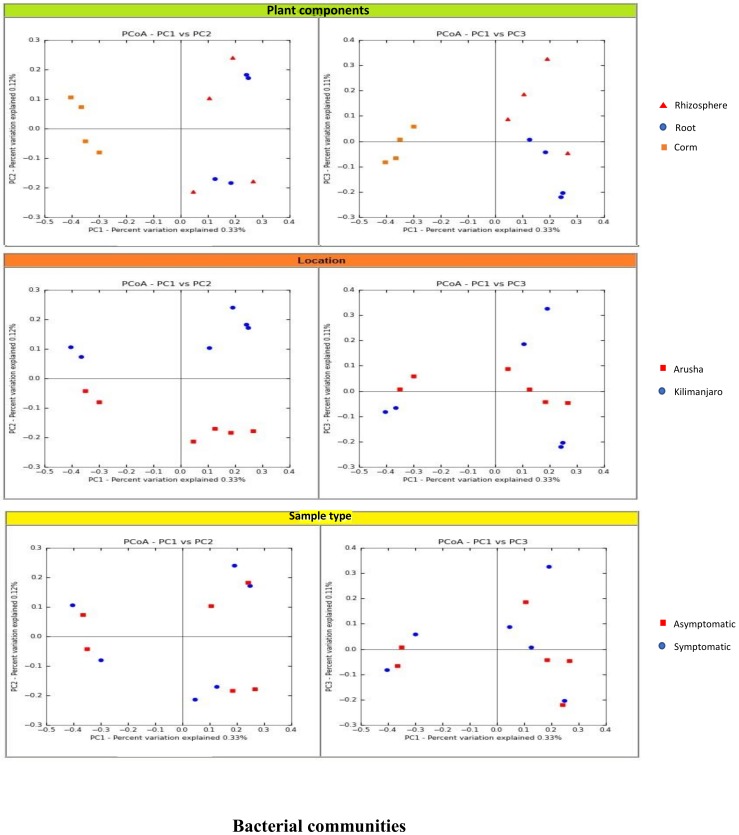

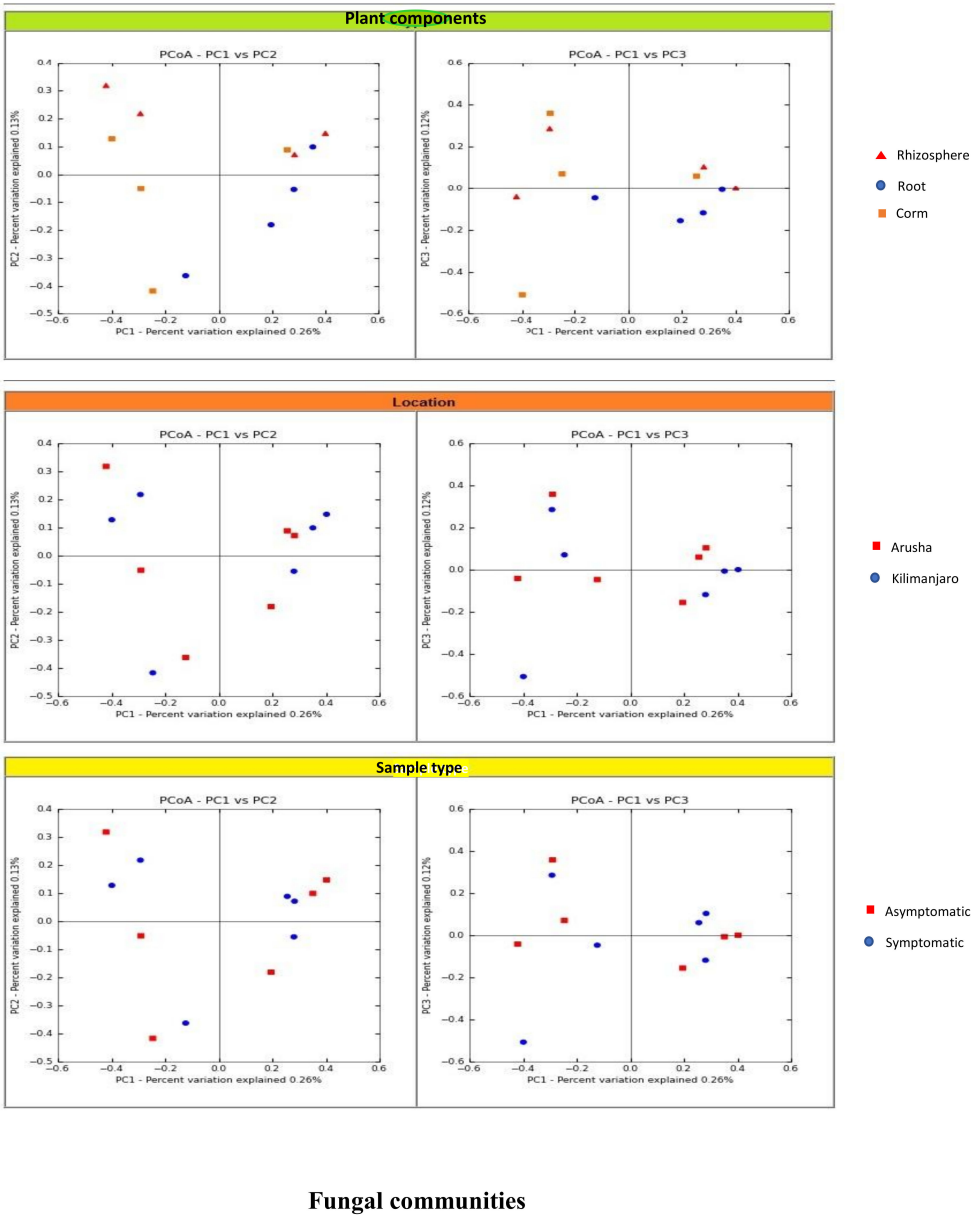
Factors driving the microbiota composition of bacterial and fungal communities in organs, locations, and sample types. The principal coordinate analyses (PCoA) of pairwise Euclidean distance matrixes of filtered operational taxonomy unit (OTU) tables. PCoA analyses were performed considering all 12 samples of 16S and ITS based on three properties, i.e., sample organ (rhizosphere, roots, and corm of banana), location (Arusha and Kilimanjaro), and sample type (asymptomatic and symptomatic). For each group, the same graph was differentially colored to emphasize the influence of sample type and location of sample in the community assemblage.

**Figure 3:**
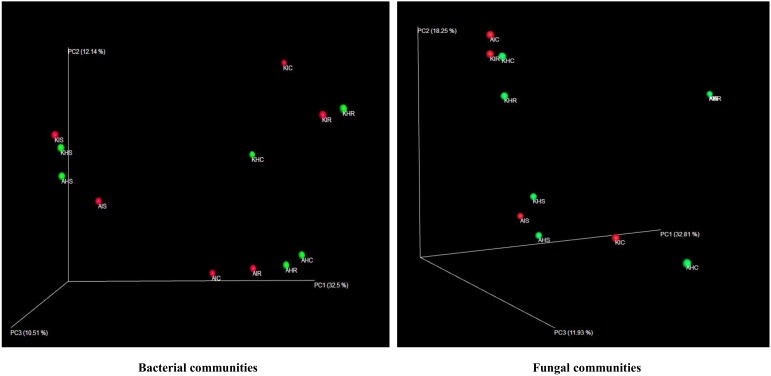
Rhizosphere, root, and corm samples of banana showing differences in their compositions and structure in bacterial and fungal communities of both locations. Principal coordinate analysis (PCoA) of pairwise, showing jackknife-supported confidence ellipsoids. The first three principal axes are shown. Principal coordinate analysis based on Euclidean distances. A: Arusha; C: Corm; H: Asymptomatic; I: Symptomatic; K: Kilimanjaro; R: Roots; S: Rhizosphere.

**Figure 4:**
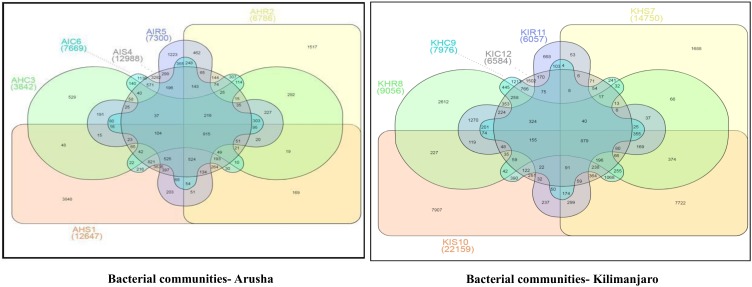

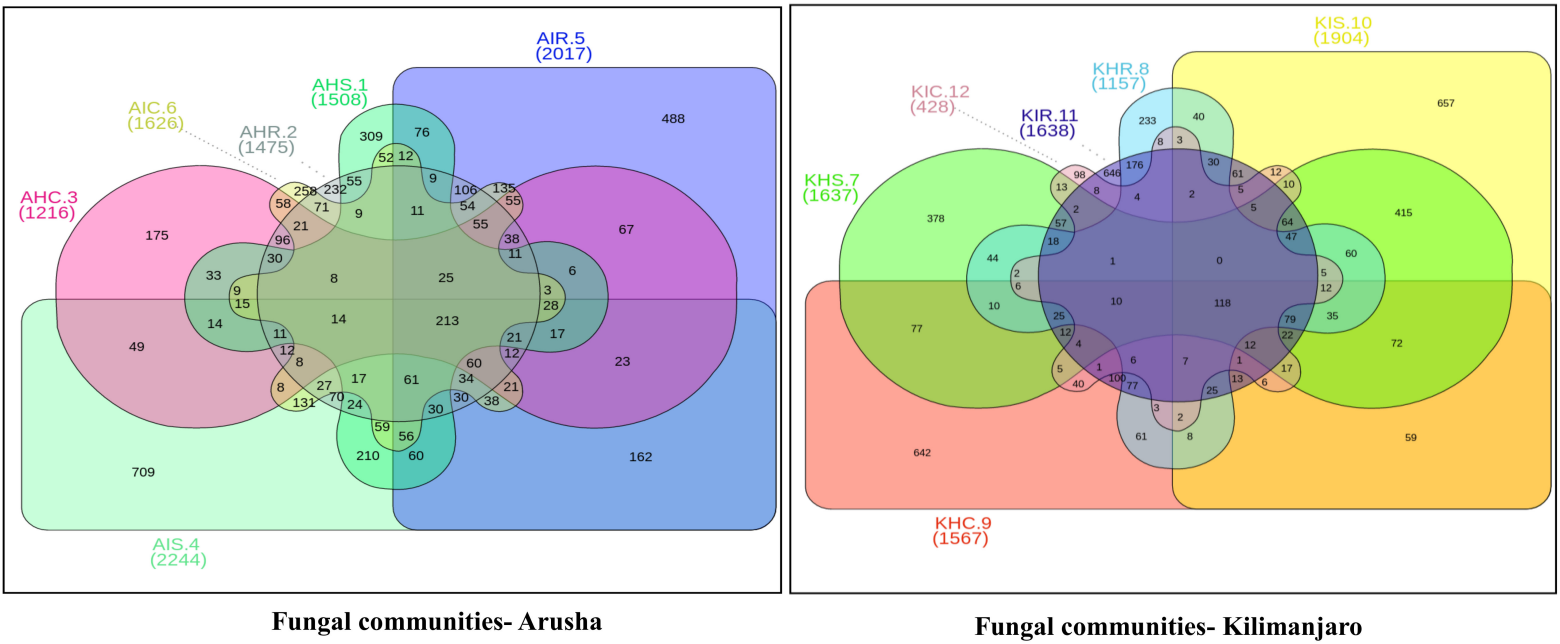
Venn diagram of the banana rhizosphere, roots, and corm. The observed OTUs for each treatment were produced in the UCLUST algorithm to show the shared and unique OTUs. Only the most abundant OTUs among all the samples were represented. A: Arusha; C: Corm; H: Asymptomatic; I: Symptomatic; K: Kilimanjaro; R: Roots; S: Rhizosphere.

We analyzed the variables such as plant component, location, and sample types (asymptomatic and symptomatic). Among the rhizosphere, root, and corm, a specific association was observed for both bacterial and fungal communities among the sample types. The rhizosphere significantly impacted the bacterial communities regardless of plant components. However, the location had a significant effect on fungal communities (Figure 2). High variability was displayed among the rhizosphere, root, and corm by the bacterial and fungal communities (Figure 3). A common pattern was observed using PCoA, with 32.50% and 32.81% differences in bacterial and fungal communities, respectively. There was not much variation observed for sample types for bacterial and fungal communities. However, a consistent trend was observed for both bacterial and fungal communities in sample types collected from the Arusha region. Operational taxonomy unit (OTU) count was maximum in the rhizosphere and was lower in the roots followed by the corm (Figure 4). A similar trend was observed for bacterial and fungal communities in sample types collected from the Kilimanjaro region. Thus, the banana rhizosphere was the major reservoir of bacterial and fungal communities in both locations. For bacterial communities, the rhizosphere displayed the greatest diversity and richness in OTUs in symptomatic (12,988 and 22,159) than in asymptomatic (12,647 and 14,750) sample types in Arusha and Kilimanjaro, respectively. An identical trend was observed for root and corm samples from Arusha, but in Kilimanjaro roots and corm, asymptomatic sample types were richer in OTUs compared to symptomatic sample types. In the case of fungal communities, highest OTU count was observed in the rhizosphere of symptomatic (2244) compared to asymptomatic (1508) sample types. This was followed by roots (2017 and 1475) and corm (1626 and 1216), respectively in the Arusha region. An identical trend was displayed for OTU richness in the rhizosphere and roots samples from Kilimanjaro. However, the OTUs of fungal communities count in corm reduced drastically in symptomatic (428) as compared to asymptomatic (1567) sample types.

We further analyzed the rhizosphere, root, and corm to know to what extent the bacterial and fungal communities were distributed and shared among these plant components. Most bacterial and fungal communities associated with the rhizosphere were also found in root and corm (Figure S2 and Table S1). *Rhizobiales, Xanthomonadales,* and *Burkholderiales* were the most dominant bacterial orders associated with each plant component in both locations for asymptomatic and symptomatic sample types. Among the fungi, *Hypocreales* was the most dominant order followed by *Agaricales, Incertae_sedis, Sordariales, Mortierellales,* and *Eurotiales* in both locations for asymptomatic and symptomatic sample types (Figure S2). As the bacterial and fungal communities shared significant proportions among different plant components, we analyzed the relative abundance of taxa. A small fraction from the total identified orders contributed to total relative abundance of both bacterial and fungal communities. It became also apparent that plant components have identical relative abundance for specific orders of bacterial and fungal communities (Figure 3).

**Figure 5:**
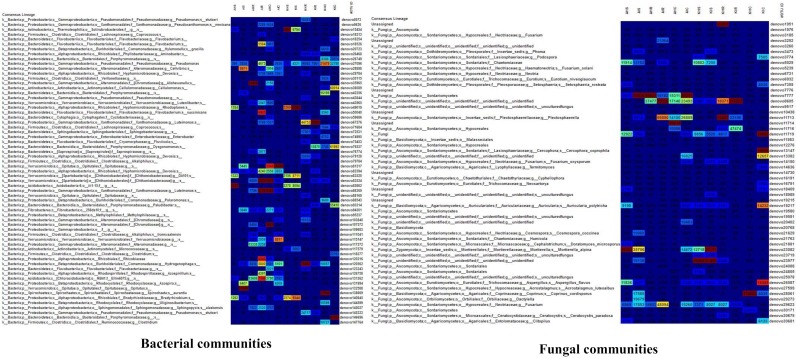
An OTU table heat map showing taxonomy assignment for each OTU from sample. The OTU heatmap displays OTU counts per sample, where the counts are colored based on the contribution of each OTU to the total OTU count present in the sample (blue: contributes low percentage of OTUs to sample; red: contributes high percentage of OTUs). The table based on taxonomy assignment is filtered the OTU table by number (10,000 for bacteria and 5,000 for fungi) of counts per OTU. A: Arusha; C: Corm; H: Asymptomatic; I: Symptomatic; K: Kilimanjaro; R: Roots; S: Rhizosphere.

In terms of bacterial communities, a relatively high abundance of *Alteromonadales* and *Burkholderiales* was found in roots in both locations in asymptomatic sample types (Figure 5). *Xanthomonadales* was the dominant order in roots in both locations in symptomatic sample types. *Spirochaetales* and *Xanthomonadales* were present in high abundance in corm in both locations in asymptomatic sample types. *Bacteroidales* and *Opitutales* were enriched in corm in both locations in symptomatic sample types. In the case of fungal communities, a large portion was recognized as “unknown”. Among the identified ones, *Mortierellales* was dominant in the rhizosphere in both locations in asymptomatic and symptomatic sample types (Figure 5). *Incertae_sedis* was the most dominant order in roots of Kilimanjaro in asymptomatic sample types. *Agaricales* and *Sordariales* were among the most abundant order in corm of Kilimanjaro asymptomatic and symptomatic sample types (Figure 5). We found a higher population of different *Fusarium* spp. including *F. oxysporum* in samples collected from symptomatic plants in both locations.

### 3.3. Bacterial and Fungal Communities Underlying Highly Abundant OTU

**Figure 6:**
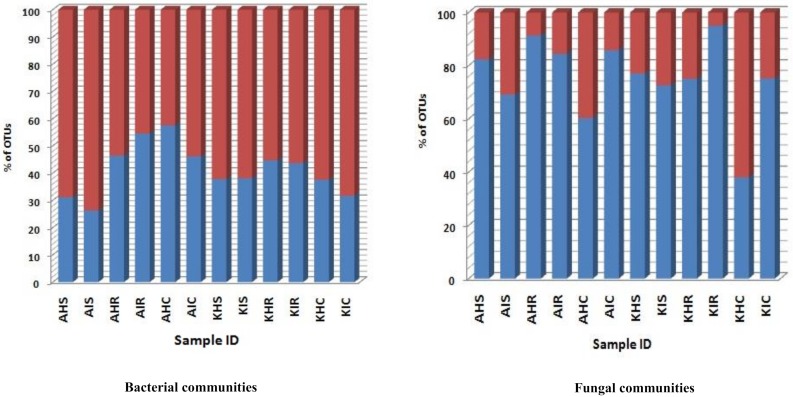
Relative abundance of OTU belonging to core and non-core OTUs. Blue bars represent relative abundance of core OTUs whereas red bars represent relative abundance of non-core OTUs. A: Arusha; C: Corm; H: Asymptomatic; I: Symptomatic; K: Kilimanjaro; R: Roots; S: Rhizosphere.

A significant level of distinct and specific genera were dominant in the rhizosphere, root, and corm in both locations and sample types. We consider that these specific bacterial and fungal communities represent the main colonizers of plant components. A low relative abundance of OTU richness was associated with bacterial communities of sample types. For fungal communities, a very high relative abundance was associated with OTU richness in sample types (Figure 6). For bacterial communities, relative abundance of OTUs in rhizosphere and corm in both locations were more in asymptomatic as compared to symptomatic sample types. An exception was found in the root samples collected in Arusha where symptomatic sample types exceeded 16% of total OTU richness compared to asymptomatic sample types (Figure 6). In case of fungal communities, OTU richness was much higher with rhizosphere and root compared to corm samples. OTU richness was higher in the rhizosphere of both locations of asymptomatic samples types. Roots and corm of Kilimanjaro of symptomatic displayed higher (20–49%) OTU richness compared to asymptomatic sample types (Figure 6).

### 3.4. Core Colonizers Among Bacterial and Fungal Communities

**Figure 7:**
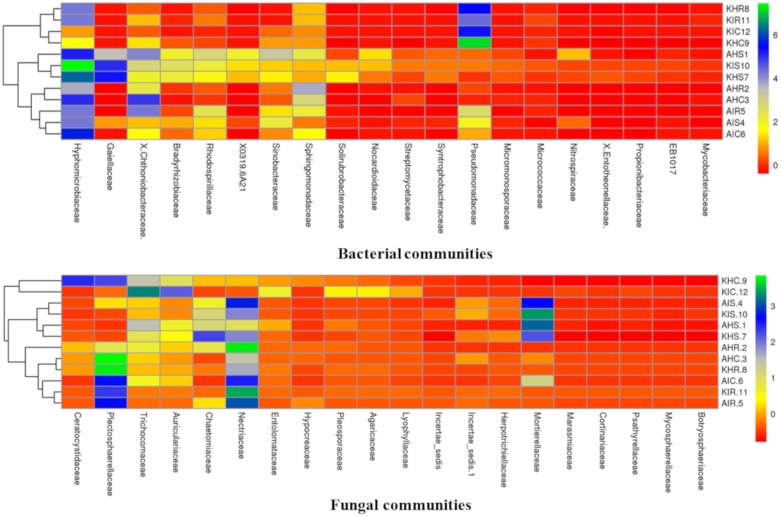
Distinct core colonizers pattern of banana samples. The heatmap display a distribution pattern of core OTUs across sample types based on relative abundance (column z-score). For plotting heatmap displaying core-colonization pattern the z-scores of relative abundance of top 20 core OTUs (family level) were considered. Samples were hierarchically grouped based on the pairwise distances. A: Arusha; C: Corm; H: Asymptomatic; I: Symptomatic; K: Kilimanjaro; R: Roots; S: Rhizosphere.

Considering the high relative abundance among samples, we analyzed core colonizers among bacterial and fungal communities. Both core bacterial and fungal communities were classified into the top 20 families that colonized distinct plant components of banana (Figure 7). Core bacterial and fungal communities displayed a unique profile in the rhizosphere, root, and corm. Fungal communities of rhizosphere and corm displayed identical profiles in both locations in sample types with preferential family colonizers. Rhizosphere, root, and corm (regardless of location and samples types) also shared communities which were more identical to each other (Figure 7).

Members of *Hyphomicrobiaceae* were found to be preferential colonizers of rhizosphere, root, and corm at both locations of sample types. *Hyphomicrobiaceae* represented 28.26% and 24.63% relative abundance in plant components in Arusha and Kilimanjaro, respectively. This was followed by *Pseudomonadaceae* (22.91%) in Kilimanjaro and *Chthoniobacteraceae* (18.36%) and *Sphingomonadaceae* (13.34%) in Arusha; both were highly abundant in sample types. In case of fungal communities, *Nectriaceae* were found to be preferential colonizers and represented 23% of the relative abundance for both locations, followed by *Plectosphaerellaceae* (17.14%), *Mortierellaceae* (9.54%), and *Trichocomaceae* (8.42%) of sample types.

Bacterial communities classified as members of *Hyphomicrobiaceae* were more abundant in the rhizosphere, and *Pseudomonadaceae* in the roots and corm in both locations of sample types. For fungal communities, *Mortierellaceae* was observed with the highest relative abundance in the rhizosphere, *Nectriaceae* in roots, and *Plectosphaerellaceae* in corm of both locations of sample types. Notably, a small group of bacterial and fungal communities were not specifically accommodated by any particular plant component. This includes core bacterial communities associated with *Chthoniobacteraceae* and *Sphingomonadaceae*, and core fungal communities were associated with *Trichocomaceae* and *Auriculariaceae*.

### 3.5. Untapped Bacterial and Fungal Communities

**Figure 8:**
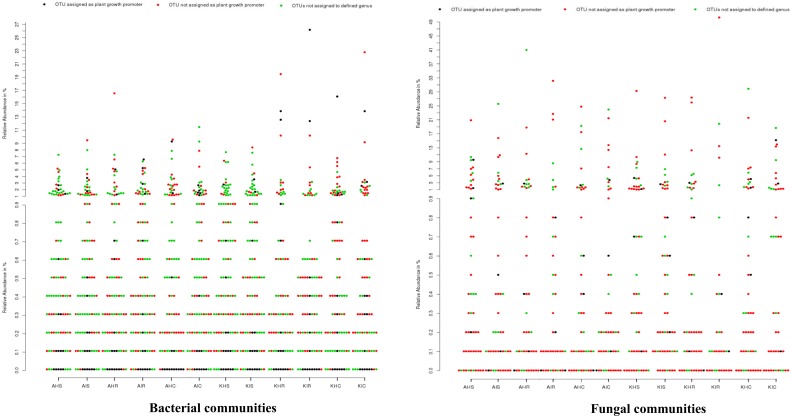
Untapped diversity in bacterial and fungal communities. The relative abundance of core OTUs is demonstrated by dots in a sample type. Dot representing OTUs where, black dots represent OTUs assigned as plant growth promoter, red dots represent OTUs unassigned as plant growth promoter, and green dots represent OTUs not assigned to a defined genus. Majority of core OTUs exhibit no functional indications in association with hosts. A: Arusha; C: Corm; H: Asymptomatic; I: Symptomatic; K: Kilimanjaro; R: Roots; S: Rhizosphere.

We analyzed the genera for plant growth promoting attributes from our identified core bacterial and fungal communities with already demonstrated genera in literature. From our core bacterial and fungal communities, 129 bacterial and 37 fungal genera could be assigned to known taxa. Among these, 15 bacterial and four fungal genera were associated with plant growth promoting traits, including *Bacillus, Pseudomonas,* and *Trichoderma* (Tables S2 and S3) plants. Other bacteria found in the rhizosphere and roots included *Bradyrhizobium, Mesorhizobium, Phyllobacterium, Rhizobium, and Azospirillum* that are known for biological nitrogen fixation; *Bacillus*, *Paenibacillus, Pseudomonas,* and *Variovorax* known to produce indole acetic acid (IAA) that is associated with promoting plant growth. The rhizosphere and root samples also included fungi belonging to *Aspergillus* and *Trichoderma,* which are known for their biocontrol attributes. Bacterial communities such as *Pseudomonadaceae*, which were supposed to be in high relative abundance in rhizosphere, root, and corm, have been poorly explored with respect to their plant growth promoting attributes in bananas. Other groups such as *Hyphomicrobiaceae* and *Sphingomonadaceae* have never been explored for their plant growth promoting traits. Regarding fungal communities, *Trichoderma* was among the highly abundant groups identified in the rhizosphere, root, and corm whose plant growth traits are known. More than 92.8% of bacterial groups that were identified have never been explored. In the case of fungal communities, very few genera (<1%) have been explored from any host plant (Figure 8).

## 4. Discussion

Banana associated bacterial and fungal communities have previously been studied by direct isolation methods. This was useful for the isolation of specific communities but limits identification of new diversity. Moreover, previous studies have focused mostly on the rhizosphere region aiming at bacterial communities with a little or no information on fungal communities. To provide a comprehensive view of the banana associated microbiome we present a critical appraisal of bacterial and fungal communities sampled in the rhizosphere, root, and corm in two different locations of asymptomatic and symptomatic sample types. We profiled the 16S and ITS2 regions to reveal the composition of bacterial and fungal communities. Our results support the concept that distinct plant components play a key role in engaging bacterial communities, irrespective of location and sample type (Figure 2).

Various plant phases have been observed to impact the rhizospheric and endophytic bacterial communities with no identical assemblage pattern [16,17,18]. The rhizosphere was the main host and passage for root colonizers to other plant components especially for bacterial communities. Bacterial communities inside a host plant originates from the rhizosphere [13]. Our study was conducted in smallholder’s fields, where banana is a perennial crop and successive crop cycle is established from suckers. Hence, through this vegetative multiplication, we assume that the sucker is automatically infected with the same bacterial and fungal communities of the preceding cycle. In addition, farmers transplant suckers from the main plant to establish new fields and thus not only possibly transfer soil borne pests and diseases but also the bacterial and fungal communities associated with the plant. Good practices, however, entail that the suckers are pared before planting to reduce nematodes and weevils [19]. Therefore, the banana corm might be the major carrier of bacterial and fungal communities in small holder farming systems. Thus, our findings support the notion of the niche-mediated host microbiome study.

We demonstrated that the rhizosphere had the greatest diversity of bacterial and fungal communities in the banana plant (Figure 2 and Figure 3) but that these communities change towards the roots and corms. Most of the bacterial and fungal communities present in roots and corm samples were also prevalent in rhizosphere samples in both locations. This suggests that bacterial and fungal communities present in roots and corm were colonized from the rhizosphere. In addition, host plants selectively promote colonization by specific bacterial and fungal communities from the rhizosphere [17,20]. It was found that the *Fusarium* wilt infected (symptomatic) samples were with a lower bacterial and fungal diversity than the asymptomatic samples. This may be due to the disease stress, which is directly linked to the decreased value of available carbon for microbes in the rhizosphere, responsible for specific microbial groups selection, and thus regulating their community compositions. Banana plant resistance and *Fusarium* aggressiveness in different plant components also contribute to bacterial and fungal prevalence. In both locations, bacterial and fungal OTU counts were maximum in the rhizosphere followed by root and corm in sample types (Figure 4). Host plants are known to support or repress colonization by certain bacterial and fungal species/genera. Thus, bacterial and fungal communities in these specific plant components either become enriched or depleted (Figure 5). Our results also displayed stable core bacterial and fungal communities (>92.8% of total relative abundance) linked with the rhizosphere, roots, and corm of sample types. We observed core bacterial and fungal communities in sample types that were preferential colonizers but differed in each plant component with respect to OTU distribution (Figure 6). Notably, these identified core bacterial and fungal communities had a higher abundance of bacterial and fungal genera specifically related to host plant growth and development [21].

A fraction of the core bacterial and fungal communities identified in the present study have previously been reported, but a larger proportion have not been reported. Some core bacterial and fungal communities were known to stimulate plant growth and promote health in various crops. We infer that these beneficial microbial communities are positively correlated with banana health. These include *Bacillus, Pseudomonas,* and some free-living diazotrophs such as *Azospirillum and Bradyrhizobium* which contribute to efficient nutrient acquisition, promote plant growth, and control soil-borne diseases. *Pseudomonas* and *Bacillus*, commonly found in the banana rhizosphere are known to promote growth, reduce stress, and act as biocontrol agents against diverse *Fusarium*-infected plant diseases [22]. Host plants may contain many diverse bacteria and fungi with the potential to enhance plant growth and confer other biological functions that benefits the plants harboring them [23,24,25]. We discovered rich and diverse fungal groups, one of them being *Aspergillus* which has already been reported for its contribution to plant growth and induced resistance against phytopathogens. Beneficial interactions of fungal communities with host plant roots are also known to improve nutrient acquisition [26,27,28,29,30]. So far, fungal communities particularly in banana received almost no attention compared to bacterial communities, but our data suggests that there is much more to discover (Figure 7). Our study demonstrates the complexity of bacterial and fungal communities which possibly interact amongst themselves (microbe–microbe) and with the host (host–microbe interactions). These cross talks might affect banana plant growth, but also result in induced resistance. It is therefore useful to study the banana microbiome in commercial plantations where banana plants are treated with inorganic fertilizers and pesticides, and compare these communities with our findings. This will allow us to study the impact of fertilizers on microbial communities as well as compare the microbial communities behaviour in commercial and small holder banana farming systems where fertilizer use is rare.

## 5. Conclusions

Our study revealed that banana plants harbor rich and diverse bacterial and fungal communities, which decreases from the rhizosphere to the roots and corm. Rhizosphere, roots, and corm components impacted bacterial communities, and different sample types influenced fungal communities distribution. Besides the high taxonomic and functional diversity in bacterial communities, we observed about 92.8% of the total relative abundance. By comparing communities in other crops, we state that several of the communities discovered in banana could serve as key nodes for plant growth and health.

## Supplementary Materials

The following are available online at https://www.mdpi.com/2076-2607/8/3/443/s1, Figure S1. Overview of sampling strategy from asymptomatic and symptomatic putative *Fusarium* infected banana plants. Each sample is a composite of three sub-samples. Figure S2. Banana rhizosphere—as a reservoir for bacterial and fungal groups that colonize plant organs. Table S2. Primers used. Table S3. Identified bacterial genera Bacterial genera described as plant-growth promoters were searched on Web of Science (http://www.webofknowledge.com). Table S4. Identified fungal genera. Fungal genera described as plant-growth promoters were searched on Web of Science (http://www.webofknowledge.com).
