# Supplementary material for: Unlocking the Microbiome Communities of Banana (Musa spp.) under Disease Stressed (Fusarium wilt) and Non-Stressed Conditions
Source: Microorganisms. 2020 Mar 20;8(3):443. doi: 10.3390/microorganisms8030443 (PMC7144012; doi:10.3390/microorganisms8030443)
Supplement: Supplementary file 1 [file microorganisms-08-00443-s001.pdf]

Supplementary Figures

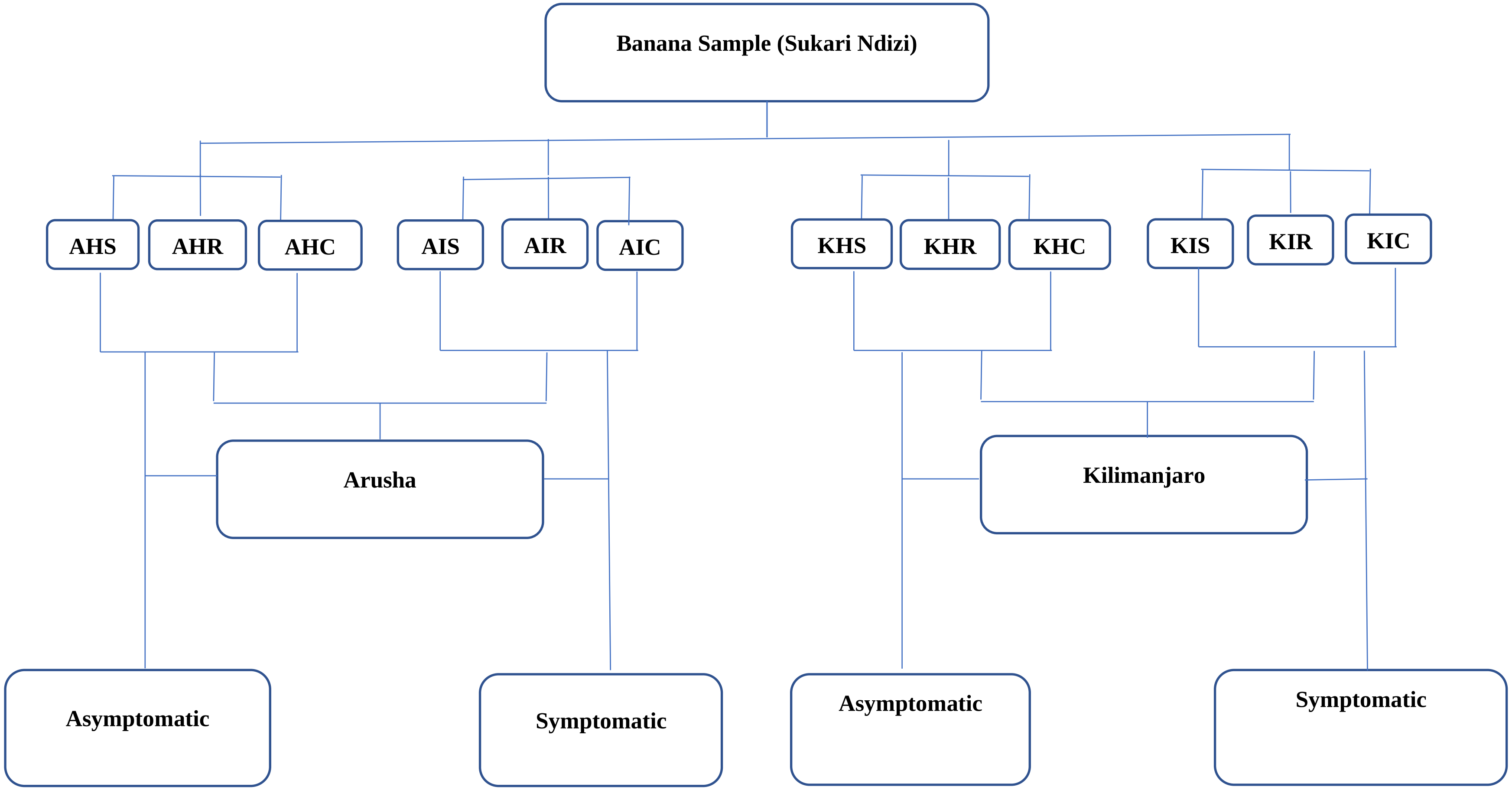

Figure S1. Overview of sampling strategy from asymptomatic and symptomatic putative *Fusarium* infected banana plants. Each sample is a composite of three sub-samples. S: Rhizosphere; R: Roots; C: Corm; A: Arusha; K: Kilimanjaro; H: Asymptomatic; I: Symptomatic

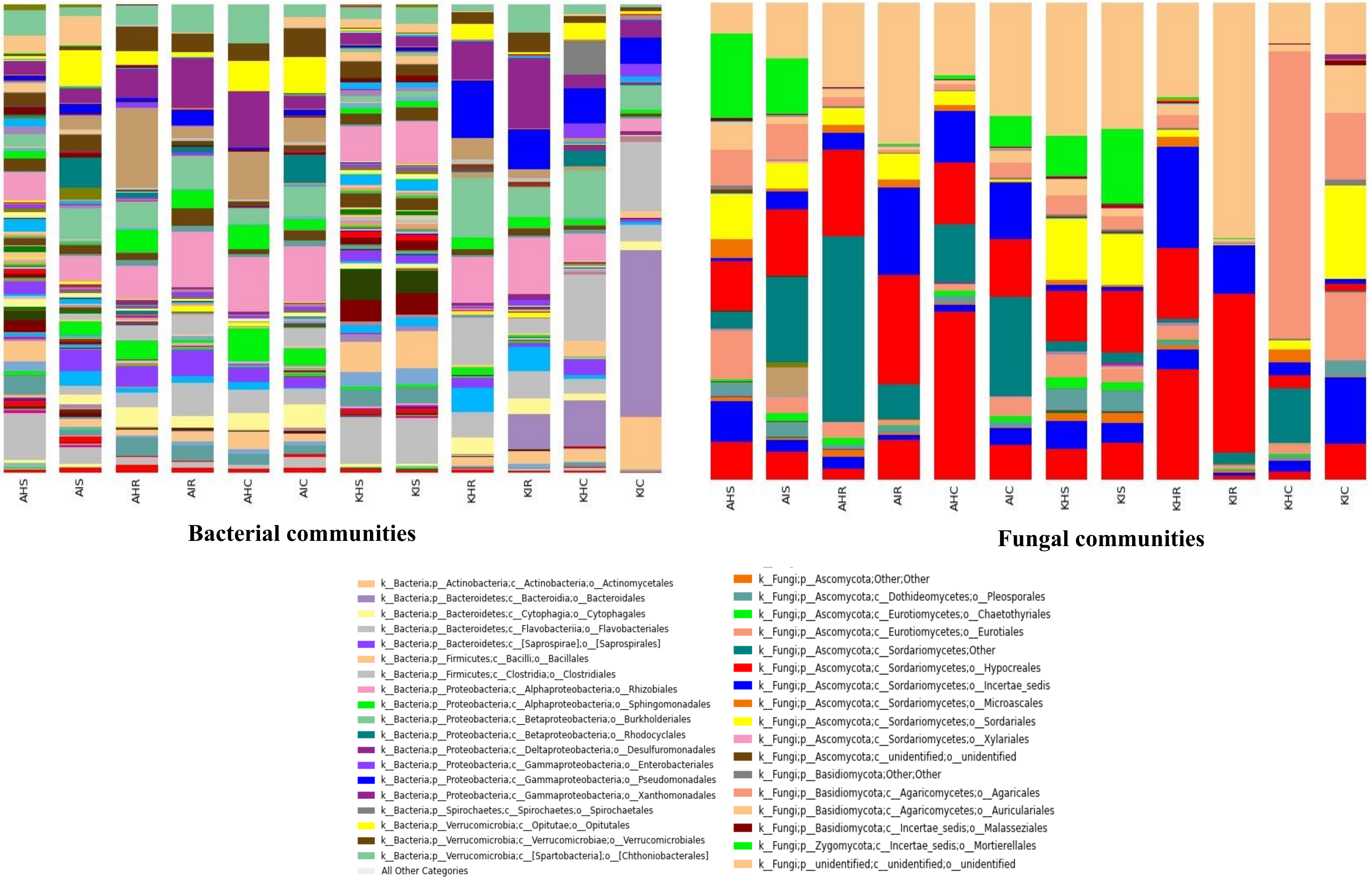
AHS
AIS
AHR
AIR
AHC
AIC
KHS
KIS
KHR
KIR
KHC
KIC
Bacterial communities
Fungal communities
k_Bacteria;p_Actinobacteria;c_Actinobacteria;o_Actinomycetales
k_Bacteria;p_Bacteroidetes;c_Bacteroidia;o_Bacteroidales
k_Bacteria;p_Bacteroidetes;c_Cytophagia;o_Cytophagales
k_Bacteria;p_Bacteroidetes;c_Flavobacteriia;o_Flavobacteriales
k_Bacteria;p_Bacteroidetes;c_[Saprospirae];o_[Saprospirales]
k_Bacteria;p_Firmicutes;c_Bacilli;o_Bacillales
k_Bacteria;p_Firmicutes;c_Clostridia;o_Clostridiales
k_Bacteria;p_Proteobacteria;c_Alphaproteobacteria;o_Rhizobiales
k_Bacteria;p_Proteobacteria;c_Alphaproteobacteria;o_Sphingomonadales
k_Bacteria;p_Proteobacteria;c_Betaproteobacteria;o_Burkholderiales
k_Bacteria;p_Proteobacteria;c_Betaproteobacteria;o_Rhodocyclales
k_Bacteria;p_Proteobacteria;c_Deltaproteobacteria;o_Desulfuromonadales
k_Bacteria;p_Proteobacteria;c_Gammaproteobacteria;o_Enterobacteriales
k_Bacteria;p_Proteobacteria;c_Gammaproteobacteria;o_Pseudomonadales
k_Bacteria;p_Proteobacteria;c_Gammaproteobacteria;o_Xanthomonadales
k_Bacteria;p_Spirochaetes;c_Spirochaetes;o_Spirochaetales
k_Bacteria;p_Verrucomicrobia;c_Opitutae;o_Opitutales
k_Bacteria;p_Verrucomicrobia;c_Verrucomicrobiae;o_Verrucomicrobiales
k_Bacteria;p_Verrucomicrobia;c_[Spartobacteria];o_[Chthoniobacterales]
All Other Categories
k_Fungi;p_Ascomycota;Other;Other
k_Fungi;p_Ascomycota;c_Dothideomycetes;o_Pleosporales
k_Fungi;p_Ascomycota;c_Eurotiomycetes;o_Chaetothyriales
k_Fungi;p_Ascomycota;c_Eurotiomycetes;o_Eurotiales
k_Fungi;p_Ascomycota;c_Sordariomycetes;Other
k_Fungi;p_Ascomycota;c_Sordariomycetes;o_Hypocreales
k_Fungi;p_Ascomycota;c_Sordariomycetes;o_Incertae_sedis
k_Fungi;p_Ascomycota;c_Sordariomycetes;o_Microascales
k_Fungi;p_Ascomycota;c_Sordariomycetes;o_Sordariales
k_Fungi;p_Ascomycota;c_Sordariomycetes;o_Xylariales
k_Fungi;p_Ascomycota;c_unidentified;o_unidentified
k_Fungi;p_Basidiomycota;Other;Other
k_Fungi;p_Basidiomycota;c_Agaricomycetes;o_Agaricales
k_Fungi;p_Basidiomycota;c_Agaricomycetes;o_Auriculariales
k_Fungi;p_Basidiomycota;c_Incertae_sedis;o_Malasseziales
k_Fungi;p_Zygomycota;c_Incertae_sedis;o_Mortierellales
k_Fungi;p_unidentified;c_unidentified;o_unidentified

Figure S2. The percentage contribution different microbial orders contribute to each category of sample. The QIIME level-4 OTU classifications (order level) were shown in figure. Numerical dataset were presented for bacterial and fungal communities (Table S1) corresponding to the percentage of bacterial and fungal communities. S: Rhizosphere; R: Roots; C: Corm; A: Arusha; K: Kilimanjaro; H: Asymptomatic; I: Symptomatic

Supplementary Tables

Table S1*:* Overview of abundant fungal order across 12 samples.

	AIS.4	KIS.10	KIR.11	AIR.5	AHR.2	AHC.3	AHS.1	KHS.7	KHR.8	AIC.6	KIC.12		
**Legend**	**Taxonomy**	**%**	**%**	**%**	**%**	**%**	**%**	**%**	**%**	**%**	**%**	**%**	**%**
k__Fungi;p__Ascomycota;c__Sordariomycetes;o__Hypocreales	2.60%	14.10%	12.90%	33.30%	22.80%	18.00%	13.00%	10.30%	10.60%	14.90%	11.90%	1.50%	
k__Fungi;p__Basidiomycota;c__Agaricomycetes;o__Agaricales	60.20%	7.40%	2.60%	0.50%	0.70%	1.90%	1.20%	7.40%	3.90%	2.60%	3.00%	13.90%	
k__Fungi;p__Ascomycota;c__Sordariomycetes;o__Incertae_sedis	2.70%	3.70%	0.70%	10.20%	18.40%	3.70%	10.80%	0.60%	1.20%	21.30%	12.00%	0.90%	
k__Fungi;p__Ascomycota;c__Sord	1.80%	5.30%	10.60%	0.20%	5.30%	3.40%	2.90%	9.30%	12.60%	1.50%	0.40%	19.60%	

ariomycetes;o__Sordariales												
k__Fungi;p__Zygomycota;c__Incertae_sedis;o__Mortierellales	**0.10%**	**11.60%**	**15.50%**	**0.00%**	**0.30%**	**0.10%**	**0.80%**	**17.80%**	**8.30%**	**0.40%**	**6.40%**	**0.00%**
k__Fungi;p__Ascomycota;c__Eurotiomycetes;o__Eurotiales	**2.00%**	**3.30%**	**3.00%**	**0.50%**	**0.90%**	**3.20%**	**1.60%**	**10.20%**	**4.80%**	**2.80%**	**4.10%**	**14.20%**

Table S1: Overview of abundant bacterial order across 12 samples.

Taxonomy	KIS10	KHS7	AHS1	KIC12	AHR2	AIS4	KHR8	KIR11	KHC9	AHC3	AIR5	AIC6
	%	%	%	%	%	%	%	%	%	%	%	%
k__Bacteria;p__Proteobacteria;c__Alphaproteobacteria;o__Rhizobiales	9.00%	7.40%	6.00%	2.70%	7.20%	5.50%	9.70%	12.00%	5.90%	11.50%	11.80%	11.80%
k__Bacteria;p__Proteobacteria;c__Gammaproteobacteria;o__Xanthomonadales	2.00%	2.50%	3.00%	3.80%	6.20%	3.10%	8.00%	14.90%	2.70%	12.00%	10.60%	2.60%
k__Bacteria;p__Proteobacteria;c__Betaproteobacteria;o__Burkholderiales	1.10%	1.30%	2.70%	5.00%	5.70%	6.70%	12.70%	6.30%	10.10%	3.50%	6.90%	6.30%
k__Bacteria;p__Firmicutes;c__Clostridia;o__Clostridiales	0.30%	0.10%	0.10%	14.90%	2.80%	1.30%	10.10%	3.10%	14.20%	0.00%	4.20%	3.80%
k__Bacteria;p__Bacteroidetes;c__Bacteroidia;o__Bacteroidales	0.20%	0.00%	0.00%	35.40%	0.00%	0.80%	0.40%	7.40%	9.80%	0.00%	0.00%	0.50%
k__Bacteria;p__Actinobacteria;c__Actinobacteria;o__Actinomycetales	7.80%	6.60%	4.50%	11.30%	1.20%	1.70%	1.90%	2.70%	2.70%	3.70%	2.20%	1.60%
k__Bacteria;p__Proteobacteria;c__Gammaproteobacteria;o__Alteromonadales	0.00%	0.10%	0.10%	0.10%	17.20%	3.00%	4.50%	1.80%	0.80%	10.30%	2.40%	5.30%
k__Bacteria;p__Verrucomicrobia;c__[Spartobacteria];o__[Chthoniobacterales]	3.60%	2.60%	5.30%	0.70%	4.20%	1.80%	1.40%	6.00%	2.10%	8.30%	6.00%	2.70%
k__Bacteria;p__Proteobacteria;c__Gammaproteobacteria;o__Pseudomonadales	0.80%	0.40%	0.80%	5.60%	0.70%	2.10%	12.20%	8.70%	7.50%	0.40%	3.30%	1.40%
k__Bacteria;p__Acidobacteria;c__Acidobacteria-6;o__iii1-15	9.80%	9.80%	10.00%	0.10%	1.70%	3.60%	0.30%	0.30%	0.50%	0.80%	1.10%	2.20%

k__Bacteria;p__Bacteroidetes;c__Flavobacteriia;o__Flavobacteriales	0.10%	0.20%	0.60%	3.60%	3.20%	1.80%	5.50%	5.80%	3.10%	5.00%	7.10%	3.30%
k__Bacteria;p__Verrucomicrobia;c__Opitutae;o__Opitutales	0.30%	0.40%	0.50%	0.60%	3.00%	7.60%	3.20%	0.50%	3.50%	6.50%	1.10%	7.70%
k__Bacteria;p__Bacteroidetes;c__[Saprospirae];o__[Saprospirales]	1.70%	2.40%	2.60%	0.60%	4.30%	4.70%	2.00%	1.00%	3.20%	3.10%	5.60%	2.20%
k__Bacteria;p__Bacteroidetes;c__Cytophagia;o__Cytophagales	0.70%	1.00%	1.60%	1.90%	4.10%	2.20%	3.40%	3.30%	1.40%	3.50%	2.50%	5.00%
k__Bacteria;p__Verrucomicrobia;c__Verrucomicrobiae;o__Verrucomicrobiales	0.20%	0.20%	0.60%	0.80%	5.10%	1.00%	2.60%	4.00%	1.70%	3.70%	4.00%	6.20%
k__Bacteria;p__Proteobacteria;c__Alphaproteobacteria;o__Sphingomonadales	1.30%	1.10%	1.60%	0.90%	4.80%	0.60%	2.40%	2.30%	1.40%	5.20%	3.90%	2.30%
k__Bacteria;p__Acidobacteria;c__[Chloracidobacteria];o__RB41	3.20%	3.60%	4.00%	0.10%	3.80%	1.10%	0.20%	0.30%	0.80%	2.30%	3.30%	1.70%
k__Bacteria;p__Proteobacteria;c__Alphaproteobacteria;o__Rhodospirillales	2.80%	2.30%	2.60%	0.60%	1.40%	2.10%	1.30%	1.60%	0.90%	0.90%	3.70%	2.20%
k__Bacteria;p__Bacteroidetes;c__Sphingobacteriia;o__Sphingobacteriales	0.20%	0.30%	0.50%	0.70%	1.00%	3.00%	5.10%	5.20%	0.90%	1.60%	1.50%	0.30%
k__Bacteria;p__Fibrobacteres;c__Fibrobacteria;o__258ds10	0.00%	0.00%	0.00%	0.00%	4.20%	2.90%	1.60%	0.50%	0.00%	7.00%	0.60%	3.60%
k__Bacteria;p__Proteobacteria;c__Betaproteobacteria;o__Rhodocyclales	0.00%	0.00%	0.40%	0.10%	1.10%	6.50%	0.40%	0.10%	3.30%	0.00%	1.30%	6.10%
k__Bacteria;p__Proteobacteria;c__Deltaproteobacteria;o__Myxococcales	2.40%	3.30%	2.70%	0.10%	0.40%	3.30%	1.00%	0.30%	0.20%	1.10%	0.80%	1.10%

k__Bacteria;p__Verrucomicrobia;c__[Pedosphaerae];o__[Pedosphaerales]	1.80%	1.70%	3.80%	0.00%	0.30%	6.30%	0.10%	0.10%	0.30%	0.10%	0.20%	2.30%
k__Bacteria;p__Actinobacteria;c__Thermoleophilia;o__Solirubrobacterales	4.80%	6.40%	2.10%	0.00%	0.10%	0.80%	0.10%	0.20%	0.10%	0.20%	0.40%	0.30%
k__Bacteria;p__Actinobacteria;c__Acidimicrobiia;o__Acidimicrobiales	3.50%	2.90%	2.10%	0.10%	0.50%	0.50%	0.20%	0.80%	0.20%	0.90%	0.80%	0.90%
k__Bacteria;p__Actinobacteria;c__Thermoleophilia;o__Gaiellales	4.60%	4.70%	2.50%	0.00%	0.10%	0.80%	0.10%	0.10%	0.10%	0.10%	0.20%	0.30%
Unassigned;Other;Other;Other	0.60%	0.90%	0.60%	0.30%	1.60%	1.00%	0.60%	0.40%	0.40%	0.90%	1.10%	1.00%
k__Bacteria;p__Firmicutes;c__Bacilli;o__Bacillales	0.60%	0.50%	1.10%	1.40%	0.30%	0.30%	0.70%	0.40%	3.50%	0.30%	0.50%	0.40%
k__Bacteria;p__Gemmatimonadetes;c__Gemmatimonadetes;o__	2.30%	2.90%	1.40%	0.00%	0.00%	0.70%	0.00%	0.30%	0.00%	0.00%	0.10%	0.00%
k__Bacteria;p__Nitrospirae;c__Nitrospira;o__Nitrospirales	2.40%	2.10%	2.70%	0.00%	0.10%	1.00%	0.00%	0.00%	0.10%	0.00%	0.20%	0.20%
k__Bacteria;p__Proteobacteria;c__Alphaproteobacteria;o__Rhodobacterales	0.20%	0.40%	0.40%	0.40%	1.30%	0.30%	0.40%	0.40%	0.80%	0.80%	1.30%	1.20%
k__Bacteria;p__Planctomycetes;c__Planctomycetia;o__Pirellulales	0.70%	0.70%	1.00%	0.00%	0.50%	0.50%	0.40%	1.20%	0.10%	0.60%	1.10%	0.80%
k__Bacteria;p__Proteobacteria;c__Gammaproteobacteria;o__Enterobacteriales	0.20%	0.00%	0.00%	2.70%	0.90%	0.00%	0.00%	0.00%	3.10%	0.00%	0.30%	0.00%
k__Bacteria;p__Spirochaetes;c__Spirochaetes;o__Spirochaetales	0.00%	0.00%	0.00%	0.00%	0.00%	0.10%	0.00%	0.00%	6.90%	0.00%	0.00%	0.20%
k__Bacteria;p__Acidobacteria;c__Solibacteres;o__Solibacterales	1.20%	0.80%	1.40%	0.10%	0.30%	1.10%	0.10%	0.00%	0.30%	0.10%	0.20%	0.60%
k__Bacteria;p__Planctomycetes;c__Phycisphaerae;o__WD2101	1.00%	0.90%	1.10%	0.10%	0.40%	0.80%	0.10%	0.10%	0.30%	0.50%	0.40%	0.80%

k__Bacteria;p__Proteobacteria;c__Alphaproteobacteria;o__Caulobacterales	0.10%	0.10%	0.00%	0.90%	1.00%	0.60%	0.60%	1.10%	0.30%	0.60%	0.30%	0.20%
k__Bacteria;p__Proteobacteria;c__Deltaproteobacteria;o__Syntrophobacterales	1.70%	1.90%	1.70%	0.00%	0.00%	0.70%	0.00%	0.00%	0.10%	0.00%	0.10%	0.20%
k__Bacteria;p__Actinobacteria;c__MB-A2-108;o__0319-7L14	1.80%	1.60%	0.90%	0.00%	0.00%	0.20%	0.00%	0.00%	0.10%	0.00%	0.00%	0.10%
k__Bacteria;p__Chloroflexi;c__Chloroflexi;o__[Roseiflexales]	2.20%	1.40%	0.70%	0.00%	0.00%	0.40%	0.00%	0.00%	0.00%	0.00%	0.00%	0.10%
k__Bacteria;p__Chloroflexi;c__Ellin6529;o__	1.20%	1.30%	0.80%	0.00%	0.00%	0.40%	0.00%	0.10%	0.00%	0.10%	0.20%	0.30%
k__Bacteria;p__Gemmatimonadetes;c__Gemm-1;o__	0.90%	1.10%	1.20%	0.00%	0.20%	0.60%	0.00%	0.00%	0.10%	0.00%	0.10%	0.30%
k__Bacteria;p__Gemmatimonadetes;c__Gemm-3;o__	0.10%	0.20%	0.00%	0.00%	0.40%	0.00%	0.60%	1.00%	0.00%	0.80%	1.20%	0.20%
k__Bacteria;p__Proteobacteria;c__Betaproteobacteria;o__	0.90%	1.20%	0.70%	0.00%	0.50%	0.40%	0.00%	0.00%	0.30%	0.20%	0.20%	0.70%
k__Bacteria;p__Proteobacteria;c__Betaproteobacteria;o__Ellin6067	0.80%	1.30%	1.60%	0.00%	0.00%	0.70%	0.00%	0.00%	0.00%	0.00%	0.00%	0.10%
k__Bacteria;p__Proteobacteria;c__Betaproteobacteria;o__MND1	1.00%	1.60%	1.70%	0.00%	0.00%	0.70%	0.00%	0.00%	0.00%	0.00%	0.00%	0.10%
k__Bacteria;p__Proteobacteria;c__Betaproteobacteria;o__SC-I-84	1.40%	0.80%	1.40%	0.00%	0.10%	0.80%	0.00%	0.00%	0.00%	0.00%	0.10%	0.30%
k__Bacteria;p__Actinobacteria;c__Actinobacteria;o__Micrococcales	1.00%	1.80%	0.30%	0.00%	0.00%	0.10%	0.00%	0.00%	0.00%	0.00%	0.00%	0.00%
k__Bacteria;p__Chloroflexi;c__Thermomicrobia;o__JG30-KF-CM45	0.60%	0.60%	0.30%	0.00%	0.20%	0.10%	0.20%	0.40%	0.10%	0.40%	0.30%	0.50%
k__Bacteria;p__Firmicutes;c__Erysipelotrichi;o__Erysipelotrichales	0.00%	0.00%	0.00%	1.10%	0.10%	0.00%	0.40%	0.20%	0.60%	0.00%	0.40%	0.30%

k__Bacteria;p__Planctomycetes;c__Planctomycetia;o__Gemmatales	0.80%	0.60%	0.80%	0.00%	0.10%	0.40%	0.00%	0.10%	0.10%	0.10%	0.30%	0.30%
k__Bacteria;p__Proteobacteria;c__Betaproteobacteria;o__Methylophilales	0.00%	0.00%	0.00%	0.10%	0.40%	0.40%	1.20%	0.10%	0.70%	0.00%	0.20%	0.50%
k__Bacteria;p__Proteobacteria;c__Gammaproteobacteria;o__	0.00%	0.00%	0.10%	0.00%	0.40%	0.10%	0.80%	0.70%	0.00%	0.40%	0.50%	0.30%
k__Bacteria;p__Acidobacteria;c__Acidobacteria-6;o__CCU21	0.50%	0.60%	0.70%	0.00%	0.00%	0.60%	0.00%	0.00%	0.00%	0.00%	0.00%	0.10%
k__Bacteria;p__Acidobacteria;c__iii1-8;o__DS-18	0.60%	0.70%	0.80%	0.00%	0.00%	0.20%	0.00%	0.00%	0.00%	0.00%	0.00%	0.00%
k__Bacteria;p__Chloroflexi;c__Anaerolineae;o__Caldilineales	0.30%	0.30%	0.20%	0.00%	0.30%	0.20%	0.20%	0.30%	0.10%	0.30%	0.30%	0.50%
k__Bacteria;p__Chloroflexi;c__S085;o__	0.40%	0.30%	0.30%	0.00%	0.20%	0.10%	0.00%	0.00%	0.00%	0.10%	0.20%	0.20%
k__Bacteria;p__Chloroflexi;c__TK10;o__B07_WMSP1	0.90%	0.80%	0.50%	0.00%	0.00%	0.20%	0.00%	0.00%	0.00%	0.00%	0.00%	0.00%
k__Bacteria;p__Gemmatimonadetes;c__Gemmatimonadetes;o__Gemmatimonadales	0.60%	0.60%	0.30%	0.00%	0.20%	0.20%	0.10%	0.20%	0.10%	0.10%	0.20%	0.00%
k__Bacteria;p__Gemmatimonadetes;c__Gemmatimonadetes;o__N1423WL	0.40%	0.50%	0.60%	0.00%	0.10%	0.20%	0.00%	0.20%	0.10%	0.00%	0.10%	0.20%
k__Bacteria;p__Planctomycetes;c__Planctomycetia;o__Planctomycetales	0.20%	0.20%	0.20%	0.00%	0.20%	0.20%	0.10%	0.10%	0.10%	0.30%	0.40%	0.60%
k__Bacteria;p__Proteobacteria;c__Alphaproteobacteria;o__Ellin329	0.30%	0.30%	0.30%	0.00%	0.10%	0.60%	0.10%	0.10%	0.20%	0.10%	0.10%	0.10%
k__Bacteria;p__Proteobacteria;c__Betaproteobacteria;o__Neisseriales	0.00%	0.00%	0.40%	0.00%	0.00%	2.50%	0.00%	0.00%	0.00%	0.00%	0.00%	0.00%

k__Bacteria;p__Proteobacteria;c__Deltaproteobacteria;o__Desulfuromonadales	0.00%	0.00%	0.00%	0.30%	0.00%	0.40%	0.30%	0.10%	1.00%	0.00%	0.00%	0.20%
k__Bacteria;p__Proteobacteria;c__Gammaproteobacteria;o__Thiotrichales	0.20%	0.30%	0.20%	0.00%	0.10%	0.20%	0.30%	0.10%	0.00%	0.10%	0.40%	0.30%
k__Bacteria;p__TM7;c__TM7-1;o__	0.30%	0.20%	0.20%	0.10%	0.20%	0.10%	0.20%	0.40%	0.10%	0.00%	0.10%	0.10%
k__Bacteria;p__WS3;c__PRR-12;o__Sediment-1	0.60%	0.50%	1.10%	0.00%	0.00%	0.50%	0.00%	0.00%	0.00%	0.00%	0.00%	0.00%
k__Bacteria;p__AD3;c__ABS-6;o__	0.20%	0.00%	0.40%	0.00%	0.00%	0.00%	0.00%	0.00%	0.00%	0.00%	0.00%	0.00%
k__Bacteria;p__Acidobacteria;c__Acidobacteria-5;o__	0.50%	0.20%	0.70%	0.00%	0.00%	0.20%	0.00%	0.00%	0.00%	0.00%	0.00%	0.00%
k__Bacteria;p__Acidobacteria;c__Acidobacteriia;o__Acidobacteriales	0.40%	0.20%	0.60%	0.00%	0.00%	0.30%	0.00%	0.00%	0.00%	0.00%	0.00%	0.00%
k__Bacteria;p__Acidobacteria;c__S035;o__	0.30%	0.20%	0.20%	0.00%	0.00%	0.10%	0.00%	0.00%	0.00%	0.00%	0.00%	0.00%
k__Bacteria;p__Acidobacteria;c__[Chloracidobacteria];o__11-24	0.40%	0.30%	0.50%	0.00%	0.00%	0.10%	0.00%	0.00%	0.00%	0.00%	0.00%	0.00%
k__Bacteria;p__Acidobacteria;c__[Chloracidobacteria];o__PK29	0.30%	0.30%	0.30%	0.00%	0.00%	0.10%	0.00%	0.00%	0.00%	0.00%	0.00%	0.00%
k__Bacteria;p__Actinobacteria;c__Rubrobacteria;o__Rubrobacterales	0.60%	0.80%	0.10%	0.00%	0.00%	0.00%	0.00%	0.00%	0.00%	0.00%	0.00%	0.00%
k__Bacteria;p__Armatimonadetes;c__Chthonomonadetes;o__SJA-22	0.30%	0.10%	0.50%	0.00%	0.00%	0.10%	0.00%	0.00%	0.00%	0.00%	0.00%	0.00%
k__Bacteria;p__Armatimonadetes;c__[Fimbriimonadia];o__[Fimbriimonadales]	0.10%	0.00%	0.10%	0.00%	0.20%	0.10%	0.10%	0.00%	0.20%	0.00%	0.00%	0.00%

k__Bacteria;p__BRC1;c__PRR-11;o__	0.00%	0.10%	0.10%	0.00%	0.20%	0.10%	0.00%	0.00%	0.00%	0.20%	0.10%	0.10%
k__Bacteria;p__Chloroflexi;c__Anaerolineae;o__SBR1031	0.10%	0.20%	0.10%	0.00%	0.10%	0.20%	0.00%	0.00%	0.10%	0.20%	0.20%	0.20%
k__Bacteria;p__Chloroflexi;c__Anaerolineae;o__envOPS12	0.10%	0.20%	0.10%	0.00%	0.00%	0.10%	0.00%	0.00%	0.00%	0.00%	0.00%	0.00%
k__Bacteria;p__Chloroflexi;c__TK10;o__AKYG885	0.30%	0.40%	0.40%	0.00%	0.00%	0.10%	0.00%	0.00%	0.00%	0.00%	0.00%	0.10%
k__Bacteria;p__Firmicutes;c__AHT28;o__	0.00%	0.00%	0.00%	0.00%	0.00%	0.00%	0.00%	0.00%	0.00%	0.00%	0.70%	0.10%
k__Bacteria;p__Gemmatimonadetes;c__Gemm-2;o__	0.00%	0.10%	0.00%	0.00%	0.50%	0.00%	0.00%	0.00%	0.00%	0.00%	0.20%	0.20%
k__Bacteria;p__Gemmatimonadetes;c__Gemmatimonadetes;o__C114	0.30%	0.30%	0.40%	0.00%	0.00%	0.10%	0.00%	0.00%	0.00%	0.00%	0.00%	0.00%
k__Bacteria;p__Gemmatimonadetes;c__Gemmatimonadetes;o__Ellin5290	0.40%	0.30%	0.20%	0.00%	0.00%	0.10%	0.00%	0.00%	0.00%	0.00%	0.00%	0.00%
k__Bacteria;p__Gemmatimonadetes;c__Gemmatimonadetes;o__KD8-87	0.00%	0.10%	0.10%	0.00%	0.20%	0.10%	0.10%	0.00%	0.20%	0.00%	0.20%	0.60%
k__Bacteria;p__NKB19;c__TSBW08;o__	0.00%	0.00%	0.00%	0.00%	0.30%	0.00%	0.10%	0.20%	0.10%	0.00%	0.00%	0.10%
k__Bacteria;p__OD1;c__ZB2;o__	0.00%	0.00%	0.00%	0.00%	0.90%	0.00%	0.00%	0.00%	0.20%	0.00%	0.00%	0.10%
k__Bacteria;p__Planctomycetes;c__Phycisphaerae;o__Phycisphaerales	0.00%	0.00%	0.10%	0.00%	0.10%	0.00%	0.00%	0.10%	0.10%	0.10%	0.10%	0.10%
k__Bacteria;p__Proteobacteria;c__Alphaproteobacteria;o__Rickettsiales	0.00%	0.00%	0.00%	0.00%	0.20%	0.10%	0.00%	0.00%	0.10%	0.00%	0.10%	0.10%
k__Bacteria;p__Proteobacteria;c__Betaproteobacteria;o__Nitrosomonadales	0.00%	0.00%	0.10%	0.00%	0.30%	0.00%	0.10%	0.10%	0.00%	0.10%	0.60%	0.10%

k__Bacteria;p__Proteobacteria;c__Deltaproteobacteria;o__	0.00%	0.00%	0.20%	0.00%	0.00%	0.10%	0.00%	0.00%	0.00%	0.00%	0.20%	0.10%
k__Bacteria;p__Proteobacteria;c__Deltaproteobacteria;o__Bdellovibrionales	0.00%	0.00%	0.10%	0.00%	0.00%	0.20%	0.10%	0.40%	0.00%	0.00%	0.00%	0.10%
k__Bacteria;p__Proteobacteria;c__Deltaproteobacteria;o__NB1-j	0.10%	0.20%	0.30%	0.00%	0.00%	0.20%	0.00%	0.00%	0.00%	0.00%	0.00%	0.00%
k__Bacteria;p__Proteobacteria;c__Deltaproteobacteria;o__[Entotheonellales]	0.60%	0.70%	0.20%	0.00%	0.00%	0.10%	0.00%	0.00%	0.00%	0.00%	0.00%	0.00%
k__Bacteria;p__Proteobacteria;c__Gammaproteobacteria;o__Aeromonadales	0.00%	0.00%	0.00%	1.00%	0.00%	0.00%	0.00%	0.00%	0.50%	0.00%	0.00%	0.00%
k__Bacteria;p__Proteobacteria;c__Gammaproteobacteria;o__Legionellales	0.10%	0.10%	0.30%	0.00%	0.10%	0.30%	0.10%	0.00%	0.10%	0.30%	0.10%	0.20%
k__Bacteria;p__TM7;c__TM7-3;o__	0.00%	0.00%	0.00%	0.00%	0.00%	0.00%	0.20%	0.30%	0.20%	0.00%	0.00%	0.00%
k__Bacteria;p__TM7;c__TM7-3;o__EW055	0.00%	0.10%	0.00%	1.00%	0.00%	0.10%	0.00%	0.00%	0.30%	0.00%	0.00%	0.00%
k__Archaea;p__Crenarchaeota;c__MBGA;o__NRP-J	0.00%	0.00%	0.00%	0.00%	0.00%	0.00%	0.00%	0.00%	0.00%	0.00%	0.00%	0.00%
k__Archaea;p__Crenarchaeota;c__MCG;o__pGrfC26	0.00%	0.00%	0.00%	0.00%	0.00%	0.00%	0.00%	0.00%	0.00%	0.00%	0.00%	0.00%
k__Archaea;p__Crenarchaeota;c__Thaumarchaeota;o__Nitrososphaerales	0.20%	0.10%	0.10%	0.00%	0.00%	0.00%	0.00%	0.00%	0.00%	0.00%	0.00%	0.00%
k__Archaea;p__Euryarchaeota;c__Methanobacteria;o__Methanobacteriales	0.00%	0.00%	0.00%	0.00%	0.00%	0.00%	0.00%	0.00%	0.00%	0.00%	0.00%	0.00%
k__Archaea;p__Euryarchaeota;c__Methanomicrobia;o__Methanomicrobiales	0.00%	0.00%	0.00%	0.00%	0.00%	0.00%	0.00%	0.00%	0.00%	0.00%	0.00%	0.00%

k__Archaea;p__Euryarchaeota;c__Methanomicrobia;o__Methanosarcinales	0.00%	0.00%	0.00%	0.00%	0.00%	0.00%	0.00%	0.00%	0.00%	0.00%	0.00%	0.00%
k__Archaea;p__Euryarchaeota;c__Thermoplasmata;o__E2	0.00%	0.00%	0.00%	0.00%	0.00%	0.00%	0.00%	0.00%	0.00%	0.00%	0.00%	0.00%
k__Archaea;p__[Parvarchaeota];c__[Parvarchaea];o__WCHD3-30	0.00%	0.00%	0.00%	0.00%	0.00%	0.00%	0.00%	0.00%	0.00%	0.00%	0.00%	0.00%
k__Archaea;p__[Parvarchaeota];c__[Parvarchaea];o__YLA114	0.00%	0.00%	0.00%	0.00%	0.00%	0.00%	0.00%	0.00%	0.00%	0.00%	0.00%	0.00%
k__Bacteria;p__;c__;o__	0.00%	0.00%	0.00%	0.00%	0.00%	0.00%	0.00%	0.00%	0.00%	0.00%	0.00%	0.00%
k__Bacteria;p__AD3;c__JG37-AG-4;o__	0.00%	0.00%	0.00%	0.00%	0.00%	0.00%	0.00%	0.00%	0.00%	0.00%	0.00%	0.00%
k__Bacteria;p__Acidobacteria;c__;o__	0.00%	0.00%	0.20%	0.00%	0.00%	0.10%	0.00%	0.00%	0.00%	0.00%	0.00%	0.00%
k__Bacteria;p__Acidobacteria;c__AT-s54;o__	0.00%	0.00%	0.00%	0.00%	0.00%	0.00%	0.00%	0.00%	0.00%	0.00%	0.00%	0.00%
k__Bacteria;p__Acidobacteria;c__Acidobacteria-6;o__BPC015	0.00%	0.00%	0.00%	0.00%	0.00%	0.00%	0.00%	0.00%	0.00%	0.00%	0.00%	0.00%
k__Bacteria;p__Acidobacteria;c__BPC102;o__	0.00%	0.00%	0.00%	0.00%	0.00%	0.00%	0.00%	0.00%	0.00%	0.00%	0.00%	0.00%
k__Bacteria;p__Acidobacteria;c__BPC102;o__MVS-40	0.10%	0.10%	0.10%	0.00%	0.00%	0.00%	0.00%	0.00%	0.00%	0.00%	0.00%	0.00%
k__Bacteria;p__Acidobacteria;c__DA052;o__Ellin6513	0.00%	0.00%	0.10%	0.00%	0.00%	0.00%	0.00%	0.00%	0.00%	0.00%	0.00%	0.00%
k__Bacteria;p__Acidobacteria;c__EC1113;o__	0.00%	0.00%	0.00%	0.00%	0.00%	0.00%	0.00%	0.00%	0.00%	0.00%	0.00%	0.00%
k__Bacteria;p__Acidobacteria;c__Holophagae;o__Holophagales	0.00%	0.00%	0.00%	0.00%	0.00%	0.40%	0.00%	0.00%	0.00%	0.00%	0.00%	0.00%
k__Bacteria;p__Acidobacteria;c__PAUC37f;o__	0.10%	0.10%	0.10%	0.00%	0.00%	0.10%	0.00%	0.00%	0.00%	0.00%	0.00%	0.00%
k__Bacteria;p__Acidobacteria;c__RB25;o__	0.00%	0.00%	0.10%	0.00%	0.00%	0.10%	0.00%	0.00%	0.00%	0.00%	0.00%	0.00%

k__Bacteria;p__Acidobacteria;c__Solibacteres;o__JH-WHS99	0.00%	0.00%	0.00%	0.00%	0.00%	0.00%	0.00%	0.00%	0.00%	0.00%	0.00%	0.00%
k__Bacteria;p__Acidobacteria;c__Sva0725;o__Sva0725	0.10%	0.10%	0.10%	0.00%	0.00%	0.10%	0.00%	0.00%	0.00%	0.00%	0.00%	0.00%
k__Bacteria;p__Acidobacteria;c__TM1;o__	0.00%	0.00%	0.00%	0.00%	0.00%	0.00%	0.00%	0.00%	0.00%	0.00%	0.00%	0.00%
k__Bacteria;p__Acidobacteria;c__[Chloracidobacteria];o__	0.00%	0.00%	0.10%	0.00%	0.00%	0.00%	0.00%	0.00%	0.00%	0.00%	0.00%	0.00%
k__Bacteria;p__Acidobacteria;c__[Chloracidobacteria];o__DS-100	0.10%	0.10%	0.20%	0.00%	0.00%	0.10%	0.00%	0.00%	0.00%	0.00%	0.00%	0.00%
k__Bacteria;p__Acidobacteria;c__[Chloracidobacteria];o__Ellin7246	0.00%	0.00%	0.00%	0.00%	0.00%	0.00%	0.00%	0.00%	0.00%	0.00%	0.00%	0.00%
k__Bacteria;p__Acidobacteria;c__iii1-8;o__	0.00%	0.00%	0.00%	0.00%	0.00%	0.00%	0.00%	0.00%	0.00%	0.00%	0.00%	0.00%
k__Bacteria;p__Acidobacteria;c__iii1-8;o__32-20	0.20%	0.10%	0.20%	0.00%	0.00%	0.00%	0.00%	0.00%	0.00%	0.00%	0.00%	0.00%
k__Bacteria;p__Acidobacteria;c__iii1-8;o__SJA-36	0.00%	0.00%	0.00%	0.00%	0.00%	0.00%	0.00%	0.00%	0.00%	0.00%	0.00%	0.00%
k__Bacteria;p__Actinobacteria;c__Actinobacteria;o__Bifidobacteriales	0.00%	0.00%	0.00%	0.00%	0.00%	0.00%	0.00%	0.00%	0.00%	0.00%	0.00%	0.00%
k__Bacteria;p__Actinobacteria;c__Coriobacteriia;o__Coriobacteriales	0.00%	0.00%	0.00%	0.00%	0.00%	0.00%	0.00%	0.00%	0.00%	0.00%	0.00%	0.00%
k__Bacteria;p__Actinobacteria;c__KIST-JJY010;o__	0.00%	0.00%	0.00%	0.00%	0.00%	0.00%	0.00%	0.00%	0.00%	0.00%	0.00%	0.00%
k__Bacteria;p__Actinobacteria;c__MB-A2-108;o__	0.10%	0.10%	0.40%	0.00%	0.00%	0.00%	0.00%	0.00%	0.00%	0.00%	0.00%	0.00%
k__Bacteria;p__Actinobacteria;c__Nitriliruptoria;o__Euzebyales	0.00%	0.00%	0.00%	0.00%	0.00%	0.00%	0.00%	0.00%	0.00%	0.00%	0.00%	0.00%

k__Bacteria;p__Actinobacteria;c__Nitriliruptoria;o__Nitriliruptorales	0.00%	0.00%	0.00%	0.00%	0.00%	0.00%	0.00%	0.00%	0.00%	0.00%	0.00%	0.00%
k__Bacteria;p__Actinobacteria;c__OPB41;o__	0.00%	0.00%	0.00%	0.00%	0.00%	0.00%	0.00%	0.00%	0.00%	0.00%	0.00%	0.00%
k__Bacteria;p__Actinobacteria;c__Thermoleophilia;o__	0.00%	0.00%	0.00%	0.00%	0.00%	0.00%	0.00%	0.00%	0.00%	0.00%	0.00%	0.00%
k__Bacteria;p__Armatimonadetes;c__0319-6E2;o__	0.00%	0.00%	0.10%	0.00%	0.00%	0.00%	0.00%	0.00%	0.00%	0.00%	0.00%	0.00%
k__Bacteria;p__Armatimonadetes;c__Armatimonadia;o__Armatimonadales	0.00%	0.00%	0.00%	0.00%	0.00%	0.00%	0.00%	0.00%	0.00%	0.00%	0.00%	0.00%
k__Bacteria;p__Armatimonadetes;c__Armatimonadia;o__FW68	0.00%	0.00%	0.00%	0.00%	0.00%	0.00%	0.00%	0.00%	0.00%	0.00%	0.00%	0.00%
k__Bacteria;p__Armatimonadetes;c__Chthonomonadetes;o__Chthonomonadales	0.10%	0.00%	0.10%	0.00%	0.00%	0.00%	0.00%	0.00%	0.00%	0.00%	0.00%	0.00%
k__Bacteria;p__Armatimonadetes;c__OPB50;o__	0.00%	0.00%	0.00%	0.00%	0.00%	0.00%	0.00%	0.00%	0.00%	0.00%	0.00%	0.00%
k__Bacteria;p__Armatimonadetes;c__SHA-37;o__	0.00%	0.00%	0.00%	0.00%	0.00%	0.00%	0.00%	0.00%	0.00%	0.00%	0.00%	0.00%
k__Bacteria;p__Armatimonadetes;c__SJA-176;o__	0.00%	0.00%	0.00%	0.00%	0.00%	0.00%	0.00%	0.00%	0.00%	0.00%	0.00%	0.00%
k__Bacteria;p__Armatimonadetes;c__SJA-176;o__GAB-B06	0.00%	0.00%	0.00%	0.00%	0.00%	0.00%	0.00%	0.00%	0.00%	0.00%	0.00%	0.00%
k__Bacteria;p__Armatimonadetes;c__SJA-176;o__RB046	0.00%	0.00%	0.00%	0.00%	0.00%	0.00%	0.00%	0.00%	0.00%	0.00%	0.00%	0.00%
k__Bacteria;p__BHI80-139;c__;o__	0.00%	0.00%	0.00%	0.00%	0.00%	0.00%	0.00%	0.00%	0.00%	0.00%	0.00%	0.00%
k__Bacteria;p__BRC1;c__NPL-UPA2;o__	0.00%	0.00%	0.00%	0.00%	0.00%	0.00%	0.00%	0.00%	0.00%	0.00%	0.00%	0.00%
k__Bacteria;p__Bacteroidetes;Other;Other	0.00%	0.00%	0.00%	0.00%	0.00%	0.00%	0.00%	0.00%	0.00%	0.00%	0.00%	0.00%

k__Bacteria;p__Bacteroidetes;c__At12OctB3;o__	0.00%	0.00%	0.00%	0.00%	0.00%	0.00%	0.10%	0.10%	0.00%	0.00%	0.00%	0.00%
k__Bacteria;p__Bacteroidetes;c__BME43;o__	0.00%	0.00%	0.00%	0.00%	0.00%	0.10%	0.00%	0.00%	0.00%	0.00%	0.00%	0.00%
k__Bacteria;p__Bacteroidetes;c__SM1A07;o__	0.00%	0.00%	0.00%	0.00%	0.00%	0.00%	0.00%	0.00%	0.00%	0.00%	0.00%	0.00%
k__Bacteria;p__Bacteroidetes;c__VC2_1_Bac22;o__	0.00%	0.00%	0.00%	0.00%	0.30%	0.00%	0.00%	0.00%	0.00%	0.00%	0.00%	0.00%
k__Bacteria;p__Bacteroidetes;c__[Rhodothermi];o__[Rhodothermales]	0.00%	0.00%	0.00%	0.00%	0.00%	0.00%	0.00%	0.00%	0.00%	0.00%	0.00%	0.00%
k__Bacteria;p__Chlamydiae;c__Chlamydiia;o__	0.00%	0.00%	0.00%	0.00%	0.00%	0.00%	0.00%	0.00%	0.00%	0.00%	0.00%	0.00%
k__Bacteria;p__Chlamydiae;c__Chlamydiia;o__Chlamydiales	0.00%	0.00%	0.00%	0.00%	0.00%	0.00%	0.00%	0.00%	0.00%	0.00%	0.00%	0.10%
k__Bacteria;p__Chlorobi;c__;o__	0.00%	0.00%	0.00%	0.00%	0.00%	0.10%	0.00%	0.00%	0.00%	0.00%	0.00%	0.00%
k__Bacteria;p__Chlorobi;c__BSV26;o__PK329	0.00%	0.00%	0.00%	0.00%	0.00%	0.00%	0.00%	0.00%	0.00%	0.00%	0.00%	0.00%
k__Bacteria;p__Chlorobi;c__BSV26;o__VC38	0.00%	0.00%	0.00%	0.00%	0.00%	0.00%	0.00%	0.00%	0.00%	0.00%	0.00%	0.00%
k__Bacteria;p__Chlorobi;c__Ignavibacteria;o__Ignavibacteriales	0.00%	0.00%	0.00%	0.00%	0.00%	0.00%	0.00%	0.00%	0.00%	0.00%	0.00%	0.00%
k__Bacteria;p__Chlorobi;c__OPB56;o__	0.00%	0.00%	0.00%	0.00%	0.00%	0.10%	0.00%	0.00%	0.00%	0.00%	0.00%	0.20%
k__Bacteria;p__Chlorobi;c__SJA-28;o__	0.00%	0.00%	0.10%	0.00%	0.10%	0.00%	0.00%	0.00%	0.00%	0.00%	0.00%	0.00%
k__Bacteria;p__Chloroflexi;Other;Other	0.00%	0.00%	0.00%	0.00%	0.00%	0.00%	0.00%	0.00%	0.00%	0.00%	0.00%	0.00%
k__Bacteria;p__Chloroflexi;c__;o__	0.10%	0.10%	0.00%	0.00%	0.00%	0.00%	0.00%	0.00%	0.00%	0.00%	0.00%	0.00%
k__Bacteria;p__Chloroflexi;c__Anaerolineae;Other	0.00%	0.00%	0.00%	0.00%	0.00%	0.00%	0.00%	0.00%	0.00%	0.00%	0.00%	0.00%

k__Bacteria;p__Chloroflexi;c__Anaerolineae;o__	0.00%	0.00%	0.00%	0.00%	0.00%	0.00%	0.00%	0.00%	0.00%	0.00%	0.00%	0.00%
k__Bacteria;p__Chloroflexi;c__Anaerolineae;o__A31	0.00%	0.00%	0.00%	0.00%	0.00%	0.00%	0.00%	0.00%	0.00%	0.00%	0.00%	0.00%
k__Bacteria;p__Chloroflexi;c__Anaerolineae;o__Anaerolineales	0.00%	0.00%	0.00%	0.00%	0.00%	0.00%	0.00%	0.00%	0.00%	0.00%	0.00%	0.00%
k__Bacteria;p__Chloroflexi;c__Anaerolineae;o__Ardenscatenales	0.00%	0.00%	0.00%	0.00%	0.00%	0.00%	0.00%	0.00%	0.00%	0.00%	0.00%	0.00%
k__Bacteria;p__Chloroflexi;c__Anaerolineae;o__CFB-26	0.00%	0.10%	0.10%	0.00%	0.00%	0.00%	0.00%	0.00%	0.00%	0.00%	0.00%	0.00%
k__Bacteria;p__Chloroflexi;c__Anaerolineae;o__DRC31	0.00%	0.00%	0.00%	0.00%	0.00%	0.00%	0.00%	0.00%	0.00%	0.00%	0.00%	0.00%
k__Bacteria;p__Chloroflexi;c__Anaerolineae;o__GCA004	0.00%	0.00%	0.00%	0.00%	0.00%	0.00%	0.00%	0.00%	0.00%	0.00%	0.00%	0.00%
k__Bacteria;p__Chloroflexi;c__Anaerolineae;o__H39	0.00%	0.00%	0.00%	0.00%	0.00%	0.00%	0.00%	0.00%	0.00%	0.00%	0.00%	0.00%
k__Bacteria;p__Chloroflexi;c__Anaerolineae;o__S0208	0.10%	0.10%	0.00%	0.00%	0.00%	0.00%	0.00%	0.00%	0.00%	0.00%	0.00%	0.00%
k__Bacteria;p__Chloroflexi;c__Anaerolineae;o__SHA-20	0.00%	0.00%	0.00%	0.00%	0.00%	0.00%	0.00%	0.00%	0.00%	0.00%	0.00%	0.00%
k__Bacteria;p__Chloroflexi;c__Anaerolineae;o__SJA-15	0.00%	0.00%	0.00%	0.00%	0.00%	0.00%	0.00%	0.00%	0.00%	0.00%	0.00%	0.00%
k__Bacteria;p__Chloroflexi;c__Anaerolineae;o__WCHB1-50	0.00%	0.00%	0.00%	0.00%	0.00%	0.00%	0.00%	0.00%	0.00%	0.00%	0.00%	0.00%
k__Bacteria;p__Chloroflexi;c__Anaerolineae;o__pLW-97	0.00%	0.00%	0.00%	0.00%	0.00%	0.00%	0.00%	0.00%	0.00%	0.00%	0.00%	0.00%
k__Bacteria;p__Chloroflexi;c__C0119;o__	0.10%	0.10%	0.10%	0.00%	0.00%	0.10%	0.00%	0.00%	0.00%	0.00%	0.00%	0.00%
k__Bacteria;p__Chloroflexi;c__Chloroflexi;o__AKIW781	0.00%	0.00%	0.00%	0.00%	0.00%	0.00%	0.00%	0.00%	0.00%	0.00%	0.00%	0.00%
k__Bacteria;p__Chloroflexi;c__Chloroflexi;o__Chloroflexales	0.00%	0.00%	0.00%	0.00%	0.00%	0.00%	0.00%	0.00%	0.00%	0.00%	0.00%	0.00%

k__Bacteria;p__Chloroflexi;c__Chloroflexi;o__Herpetosiphonales	0.00%	0.00%	0.00%	0.00%	0.00%	0.20%	0.00%	0.00%	0.00%	0.00%	0.00%	0.00%
k__Bacteria;p__Chloroflexi;c__Dehalococcoidetes;Other	0.00%	0.00%	0.00%	0.00%	0.00%	0.00%	0.00%	0.00%	0.00%	0.00%	0.00%	0.00%
k__Bacteria;p__Chloroflexi;c__Dehalococcoidetes;o__Dehalococcoidales	0.00%	0.00%	0.00%	0.00%	0.00%	0.00%	0.00%	0.00%	0.00%	0.00%	0.00%	0.00%
k__Bacteria;p__Chloroflexi;c__Gitt-GS-136;o__	0.10%	0.20%	0.10%	0.00%	0.00%	0.00%	0.00%	0.00%	0.00%	0.00%	0.00%	0.00%
k__Bacteria;p__Chloroflexi;c__Ktedonobacteria;o__B12-WMSP1	0.00%	0.00%	0.00%	0.00%	0.00%	0.00%	0.00%	0.00%	0.00%	0.00%	0.00%	0.00%
k__Bacteria;p__Chloroflexi;c__Ktedonobacteria;o__JG30-KF-AS9	0.10%	0.00%	0.10%	0.00%	0.00%	0.00%	0.00%	0.00%	0.00%	0.00%	0.00%	0.00%
k__Bacteria;p__Chloroflexi;c__Ktedonobacteria;o__Ktedonobacterales	0.00%	0.00%	0.10%	0.00%	0.00%	0.10%	0.00%	0.00%	0.00%	0.00%	0.00%	0.00%
k__Bacteria;p__Chloroflexi;c__Ktedonobacteria;o__Thermogemmatisporales	0.10%	0.00%	0.10%	0.00%	0.00%	0.00%	0.00%	0.00%	0.00%	0.00%	0.00%	0.00%
k__Bacteria;p__Chloroflexi;c__P2-11E;o__	0.00%	0.00%	0.10%	0.00%	0.00%	0.00%	0.00%	0.00%	0.00%	0.00%	0.00%	0.00%
k__Bacteria;p__Chloroflexi;c__SAR202;o__	0.00%	0.00%	0.00%	0.00%	0.00%	0.00%	0.00%	0.00%	0.00%	0.00%	0.00%	0.00%
k__Bacteria;p__Chloroflexi;c__SHA-26;o__	0.20%	0.20%	0.10%	0.00%	0.00%	0.10%	0.00%	0.00%	0.00%	0.00%	0.00%	0.00%
k__Bacteria;p__Chloroflexi;c__TK10;o__	0.10%	0.20%	0.10%	0.00%	0.00%	0.00%	0.00%	0.00%	0.00%	0.00%	0.00%	0.00%
k__Bacteria;p__Chloroflexi;c__TK17;o__	0.00%	0.10%	0.00%	0.00%	0.00%	0.00%	0.00%	0.10%	0.00%	0.00%	0.00%	0.00%
k__Bacteria;p__Chloroflexi;c__TK17;o__mle1-48	0.10%	0.10%	0.00%	0.00%	0.00%	0.00%	0.00%	0.00%	0.00%	0.00%	0.00%	0.10%
k__Bacteria;p__Chloroflexi;c__Thermomicrobia;o__	0.00%	0.00%	0.00%	0.00%	0.00%	0.00%	0.00%	0.00%	0.00%	0.00%	0.00%	0.00%

k__Bacteria;p__Chloroflexi;c__Thermomicrobia;o__AKYG1722	0.10%	0.00%	0.00%	0.00%	0.00%	0.00%	0.10%	0.10%	0.00%	0.00%	0.00%	0.00%
k__Bacteria;p__Chloroflexi;c__Thermomicrobia;o__Ellin6537	0.00%	0.00%	0.00%	0.00%	0.00%	0.00%	0.00%	0.00%	0.00%	0.00%	0.00%	0.00%
k__Bacteria;p__Chloroflexi;c__Thermomicrobia;o__Sphaerobacterales	0.00%	0.00%	0.00%	0.00%	0.00%	0.00%	0.00%	0.00%	0.00%	0.00%	0.00%	0.00%
k__Bacteria;p__Cyanobacteria;c__4C0d-2;o__MLE1-12	0.00%	0.00%	0.00%	0.00%	0.00%	0.00%	0.00%	0.00%	0.00%	0.00%	0.00%	0.00%
k__Bacteria;p__Cyanobacteria;c__4C0d-2;o__SM1D11	0.00%	0.00%	0.00%	0.00%	0.00%	0.00%	0.00%	0.00%	0.00%	0.00%	0.00%	0.00%
k__Bacteria;p__Cyanobacteria;c__4C0d-2;o__YS2	0.00%	0.00%	0.00%	0.00%	0.00%	0.00%	0.00%	0.00%	0.00%	0.00%	0.00%	0.00%
k__Bacteria;p__Cyanobacteria;c__Chloroplast;o__	0.00%	0.00%	0.00%	0.00%	0.00%	0.00%	0.00%	0.00%	0.00%	0.00%	0.00%	0.00%
k__Bacteria;p__Cyanobacteria;c__Chloroplast;o__Chlorophyta	0.00%	0.00%	0.00%	0.00%	0.00%	0.00%	0.00%	0.00%	0.00%	0.10%	0.00%	0.00%
k__Bacteria;p__Cyanobacteria;c__Chloroplast;o__Stramenopiles	0.00%	0.00%	0.00%	0.00%	0.10%	0.00%	0.00%	0.00%	0.00%	0.00%	0.00%	0.00%
k__Bacteria;p__Cyanobacteria;c__Chloroplast;o__Streptophyta	0.00%	0.00%	0.00%	0.10%	0.00%	0.00%	0.00%	0.00%	0.00%	0.00%	0.00%	0.00%
k__Bacteria;p__Cyanobacteria;c__ML635J-21;o__	0.00%	0.00%	0.00%	0.00%	0.00%	0.00%	0.00%	0.00%	0.00%	0.00%	0.00%	0.00%
k__Bacteria;p__Cyanobacteria;c__Nostocophycideae;o__Nostocales	0.00%	0.00%	0.00%	0.00%	0.00%	0.00%	0.00%	0.00%	0.00%	0.00%	0.00%	0.00%
k__Bacteria;p__Cyanobacteria;c__Oscillatoriophycideae;o__Chroococcales	0.00%	0.00%	0.00%	0.00%	0.00%	0.00%	0.00%	0.00%	0.00%	0.00%	0.00%	0.00%
k__Bacteria;p__Cyanobacteria;c__Oscillatoriophycideae;o__Oscillatoriales	0.00%	0.00%	0.00%	0.00%	0.00%	0.00%	0.00%	0.00%	0.00%	0.00%	0.00%	0.00%

k__Bacteria;p__Cyanobacteria;c__Synechococcophycideae;o__Pseudanabaenales	0.00%	0.00%	0.00%	0.00%	0.00%	0.00%	0.00%	0.00%	0.00%	0.00%	0.00%	0.00%
k__Bacteria;p__Cyanobacteria;c__Synechococcophycideae;o__Synechococcales	0.00%	0.00%	0.00%	0.00%	0.00%	0.00%	0.00%	0.00%	0.00%	0.00%	0.00%	0.00%
k__Bacteria;p__Elusimicrobia;c__Elusimicrobia;o__Elusimicrobiales	0.00%	0.00%	0.00%	0.00%	0.00%	0.00%	0.00%	0.00%	0.00%	0.00%	0.00%	0.00%
k__Bacteria;p__Elusimicrobia;c__Elusimicrobia;o__FAC88	0.10%	0.00%	0.00%	0.00%	0.00%	0.00%	0.00%	0.00%	0.00%	0.00%	0.00%	0.00%
k__Bacteria;p__Elusimicrobia;c__Elusimicrobia;o__IIb	0.00%	0.00%	0.00%	0.00%	0.00%	0.00%	0.00%	0.00%	0.00%	0.00%	0.00%	0.00%
k__Bacteria;p__Elusimicrobia;c__Elusimicrobia;o__MVP-88	0.00%	0.00%	0.00%	0.00%	0.00%	0.00%	0.00%	0.00%	0.00%	0.00%	0.00%	0.00%
k__Bacteria;p__Elusimicrobia;c__Endomicrobia;o__	0.00%	0.00%	0.00%	0.00%	0.00%	0.00%	0.00%	0.00%	0.00%	0.00%	0.00%	0.00%
k__Bacteria;p__FBP;c__;o__	0.00%	0.00%	0.00%	0.00%	0.10%	0.00%	0.00%	0.00%	0.00%	0.10%	0.00%	0.00%
k__Bacteria;p__FCPU426;c__;o__	0.00%	0.00%	0.00%	0.00%	0.00%	0.10%	0.00%	0.00%	0.00%	0.00%	0.00%	0.00%
k__Bacteria;p__Fibrobacteres;c__TG3;o__TG3-1	0.00%	0.00%	0.00%	0.00%	0.00%	0.00%	0.00%	0.00%	0.00%	0.00%	0.00%	0.00%
k__Bacteria;p__Firmicutes;c__Bacilli;o__Haloplasmatales	0.00%	0.00%	0.00%	0.00%	0.00%	0.00%	0.00%	0.00%	0.00%	0.00%	0.00%	0.00%
k__Bacteria;p__Firmicutes;c__Bacilli;o__Lactobacillales	0.00%	0.00%	0.00%	0.00%	0.00%	0.00%	0.00%	0.00%	0.00%	0.00%	0.00%	0.00%
k__Bacteria;p__Firmicutes;c__Bacilli;o__Turicibacterales	0.00%	0.00%	0.00%	0.00%	0.00%	0.00%	0.00%	0.00%	0.00%	0.00%	0.00%	0.00%
k__Bacteria;p__Firmicutes;c__Clostridia;o__Halanaerobiales	0.00%	0.00%	0.00%	0.00%	0.00%	0.00%	0.00%	0.00%	0.00%	0.00%	0.00%	0.00%
k__Bacteria;p__Firmicutes;c__Clostridia;o__MBA08	0.00%	0.00%	0.00%	0.00%	0.00%	0.00%	0.00%	0.10%	0.00%	0.00%	0.00%	0.00%
k__Bacteria;p__Firmicutes;c__Clostridia;o__Natranaerobiales	0.00%	0.00%	0.00%	0.00%	0.00%	0.00%	0.00%	0.00%	0.00%	0.00%	0.00%	0.00%

k__Bacteria;p__Firmicutes;c__Clostridia;o__OPB54	0.00%	0.00%	0.00%	0.00%	0.00%	0.00%	0.00%	0.00%	0.00%	0.00%	0.00%	0.00%
k__Bacteria;p__Firmicutes;c__Clostridia;o__Thermoanaerobacterales	0.00%	0.00%	0.00%	0.00%	0.00%	0.00%	0.00%	0.00%	0.00%	0.00%	0.00%	0.00%
k__Bacteria;p__Fusobacteria;c__Fusobacteriia;o__Fusobacteriales	0.00%	0.00%	0.00%	0.00%	0.00%	0.00%	0.00%	0.00%	0.00%	0.00%	0.00%	0.00%
k__Bacteria;p__GAL15;c__;o__	0.00%	0.00%	0.00%	0.00%	0.00%	0.00%	0.00%	0.00%	0.00%	0.00%	0.00%	0.00%
k__Bacteria;p__GN02;c__3BR-5F;o__	0.00%	0.00%	0.00%	0.00%	0.00%	0.00%	0.00%	0.00%	0.00%	0.00%	0.00%	0.00%
k__Bacteria;p__GN02;c__BD1-5;o__	0.00%	0.00%	0.00%	0.00%	0.00%	0.00%	0.00%	0.00%	0.00%	0.00%	0.00%	0.00%
k__Bacteria;p__GN02;c__GKS2-174;o__	0.00%	0.00%	0.00%	0.00%	0.00%	0.00%	0.00%	0.00%	0.00%	0.00%	0.00%	0.00%
k__Bacteria;p__GN04;c__MSB-5A5;o__	0.00%	0.00%	0.00%	0.00%	0.00%	0.00%	0.00%	0.00%	0.00%	0.00%	0.00%	0.00%
k__Bacteria;p__Gemmatimonadetes;c__;o__	0.00%	0.00%	0.00%	0.00%	0.00%	0.00%	0.00%	0.00%	0.00%	0.00%	0.00%	0.00%
k__Bacteria;p__Gemmatimonadetes;c__Gemm-4;o__	0.00%	0.00%	0.00%	0.00%	0.00%	0.00%	0.00%	0.00%	0.00%	0.00%	0.00%	0.00%
k__Bacteria;p__Gemmatimonadetes;c__Gemm-5;o__	0.10%	0.10%	0.20%	0.00%	0.00%	0.00%	0.00%	0.00%	0.00%	0.00%	0.10%	0.00%
k__Bacteria;p__Gemmatimonadetes;c__Gemmatimonadetes;Other	0.00%	0.10%	0.00%	0.00%	0.00%	0.00%	0.00%	0.00%	0.00%	0.00%	0.00%	0.00%
k__Bacteria;p__Kazan-3B-28;c__;o__	0.00%	0.00%	0.00%	0.00%	0.00%	0.00%	0.00%	0.00%	0.00%	0.00%	0.00%	0.00%
k__Bacteria;p__Lentisphaerae;c__[Lentisphaeria];o__Victivallales	0.00%	0.00%	0.00%	0.00%	0.00%	0.00%	0.00%	0.00%	0.00%	0.00%	0.00%	0.00%
k__Bacteria;p__MVP-21;c__;o__	0.00%	0.00%	0.00%	0.00%	0.00%	0.00%	0.00%	0.00%	0.00%	0.00%	0.00%	0.00%
k__Bacteria;p__NKB19;c__;o__	0.00%	0.00%	0.00%	0.00%	0.00%	0.00%	0.00%	0.00%	0.00%	0.00%	0.00%	0.00%
k__Bacteria;p__OD1;c__;o__	0.00%	0.00%	0.00%	0.00%	0.10%	0.00%	0.00%	0.00%	0.00%	0.00%	0.00%	0.00%

k__Bacteria;p__OD1;c__ABY1;o__	0.00%	0.00%	0.00%	0.00%	0.00%	0.00%	0.00%	0.00%	0.00%	0.00%	0.00%	0.00%
k__Bacteria;p__OD1;c__Mb-NB09;o__	0.00%	0.00%	0.00%	0.00%	0.00%	0.10%	0.00%	0.00%	0.00%	0.00%	0.00%	0.00%
k__Bacteria;p__OD1;c__SM2F11;o__	0.00%	0.00%	0.00%	0.00%	0.10%	0.00%	0.00%	0.00%	0.00%	0.00%	0.00%	0.10%
k__Bacteria;p__OP11;c__;o__	0.00%	0.00%	0.00%	0.00%	0.00%	0.00%	0.10%	0.20%	0.00%	0.00%	0.00%	0.00%
k__Bacteria;p__OP11;c__OP11-3;o__	0.00%	0.00%	0.00%	0.00%	0.00%	0.00%	0.00%	0.00%	0.00%	0.00%	0.00%	0.00%
k__Bacteria;p__OP11;c__OP11-4;o__	0.00%	0.00%	0.00%	0.00%	0.00%	0.00%	0.00%	0.00%	0.00%	0.00%	0.00%	0.00%
k__Bacteria;p__OP11;c__WCHB1-64;o__	0.00%	0.00%	0.00%	0.00%	0.00%	0.00%	0.00%	0.00%	0.00%	0.00%	0.00%	0.00%
k__Bacteria;p__OP11;c__WCHB1-64;o__K2-4-19	0.00%	0.00%	0.00%	0.00%	0.00%	0.00%	0.00%	0.00%	0.00%	0.00%	0.00%	0.00%
k__Bacteria;p__OP11;c__WCHB1-64;o__d153	0.00%	0.00%	0.00%	0.00%	0.10%	0.10%	0.00%	0.00%	0.00%	0.00%	0.00%	0.00%
k__Bacteria;p__OP3;c__PBS-25;o__	0.00%	0.00%	0.00%	0.00%	0.00%	0.00%	0.00%	0.00%	0.00%	0.00%	0.00%	0.00%
k__Bacteria;p__Planctomycetes;c__;o__	0.00%	0.00%	0.00%	0.00%	0.00%	0.00%	0.00%	0.00%	0.00%	0.00%	0.00%	0.00%
k__Bacteria;p__Planctomycetes;c__028H05-P-BN-P5;o__	0.00%	0.00%	0.00%	0.00%	0.00%	0.00%	0.00%	0.00%	0.00%	0.00%	0.00%	0.00%
k__Bacteria;p__Planctomycetes;c__BD7-11;o__	0.00%	0.00%	0.00%	0.00%	0.00%	0.00%	0.00%	0.00%	0.00%	0.00%	0.00%	0.00%
k__Bacteria;p__Planctomycetes;c__C6;o__MVS-107	0.00%	0.00%	0.00%	0.00%	0.00%	0.00%	0.00%	0.00%	0.00%	0.00%	0.00%	0.00%
k__Bacteria;p__Planctomycetes;c__C6;o__d113	0.00%	0.00%	0.00%	0.00%	0.00%	0.00%	0.00%	0.00%	0.00%	0.00%	0.00%	0.00%
k__Bacteria;p__Planctomycetes;c__OM190;o__	0.00%	0.00%	0.00%	0.00%	0.00%	0.00%	0.00%	0.00%	0.00%	0.00%	0.00%	0.00%

k__Bacteria;p__Planctomycetes;c__OM190;o__CL500-15	0.00%	0.00%	0.00%	0.00%	0.00%	0.00%	0.00%	0.00%	0.00%	0.00%	0.00%	0.00%
k__Bacteria;p__Planctomycetes;c__OM190;o__agg27	0.00%	0.00%	0.00%	0.00%	0.00%	0.00%	0.00%	0.00%	0.00%	0.00%	0.00%	0.00%
k__Bacteria;p__Planctomycetes;c__Phycisphaerae;o__CCM11a	0.00%	0.00%	0.00%	0.00%	0.00%	0.00%	0.00%	0.00%	0.00%	0.00%	0.00%	0.00%
k__Bacteria;p__Planctomycetes;c__Phycisphaerae;o__CPla-3	0.00%	0.00%	0.00%	0.00%	0.00%	0.10%	0.00%	0.00%	0.00%	0.00%	0.00%	0.00%
k__Bacteria;p__Planctomycetes;c__Phycisphaerae;o__Pla1	0.00%	0.00%	0.00%	0.00%	0.00%	0.00%	0.00%	0.00%	0.00%	0.00%	0.00%	0.00%
k__Bacteria;p__Planctomycetes;c__Phycisphaerae;o__S-70	0.00%	0.00%	0.00%	0.00%	0.00%	0.00%	0.00%	0.00%	0.00%	0.00%	0.00%	0.00%
k__Bacteria;p__Planctomycetes;c__Phycisphaerae;o__mle1-8	0.00%	0.00%	0.00%	0.00%	0.00%	0.00%	0.00%	0.00%	0.00%	0.00%	0.00%	0.00%
k__Bacteria;p__Planctomycetes;c__Pla3;o__	0.00%	0.00%	0.00%	0.00%	0.00%	0.00%	0.00%	0.00%	0.00%	0.00%	0.00%	0.00%
k__Bacteria;p__Planctomycetes;c__Pla4;o__	0.00%	0.00%	0.00%	0.00%	0.00%	0.00%	0.00%	0.00%	0.00%	0.00%	0.00%	0.00%
k__Bacteria;p__Planctomycetes;c__Planctomycetia;o__B97	0.00%	0.00%	0.00%	0.00%	0.00%	0.00%	0.00%	0.00%	0.00%	0.00%	0.00%	0.00%
k__Bacteria;p__Planctomycetes;c__vadinHA49;o__DH61	0.00%	0.00%	0.00%	0.00%	0.00%	0.00%	0.00%	0.00%	0.00%	0.00%	0.00%	0.00%
k__Bacteria;p__Planctomycetes;c__vadinHA49;o__PeHg47	0.00%	0.00%	0.00%	0.00%	0.00%	0.00%	0.00%	0.00%	0.00%	0.00%	0.00%	0.00%
k__Bacteria;p__Planctomycetes;c__vadinHA49;o__p04_C01	0.00%	0.00%	0.00%	0.00%	0.00%	0.00%	0.00%	0.00%	0.00%	0.00%	0.00%	0.00%
k__Bacteria;p__Proteobacteria;Other;Other	0.00%	0.00%	0.00%	0.00%	0.00%	0.00%	0.00%	0.00%	0.00%	0.00%	0.00%	0.00%
k__Bacteria;p__Proteobacteria;c__;o__	0.00%	0.00%	0.00%	0.00%	0.00%	0.10%	0.00%	0.00%	0.00%	0.00%	0.00%	0.00%
k__Bacteria;p__Proteobacteria;c__Alphaproteobacteria;Other	0.00%	0.00%	0.00%	0.00%	0.00%	0.00%	0.00%	0.00%	0.00%	0.00%	0.00%	0.00%

k__Bacteria;p__Proteobacteria;c__Alphaproteobacteria;o__	0.00%	0.10%	0.10%	0.00%	0.10%	0.10%	0.00%	0.00%	0.00%	0.00%	0.00%	0.00%
k__Bacteria;p__Proteobacteria;c__Alphaproteobacteria;o__BD7-3	0.00%	0.00%	0.00%	0.00%	0.10%	0.10%	0.00%	0.00%	0.10%	0.00%	0.10%	0.10%
k__Bacteria;p__Proteobacteria;c__Alphaproteobacteria;o__Kiloniellales	0.00%	0.00%	0.00%	0.00%	0.00%	0.00%	0.00%	0.00%	0.00%	0.00%	0.00%	0.00%
k__Bacteria;p__Proteobacteria;c__Betaproteobacteria;Other	0.00%	0.00%	0.00%	0.00%	0.00%	0.00%	0.00%	0.00%	0.00%	0.00%	0.00%	0.00%
k__Bacteria;p__Proteobacteria;c__Betaproteobacteria;o__A21b	0.10%	0.10%	0.20%	0.00%	0.00%	0.00%	0.00%	0.00%	0.00%	0.00%	0.00%	0.00%
k__Bacteria;p__Proteobacteria;c__Betaproteobacteria;o__Gallionellales	0.00%	0.00%	0.00%	0.00%	0.00%	0.00%	0.00%	0.00%	0.00%	0.00%	0.00%	0.00%
k__Bacteria;p__Proteobacteria;c__Betaproteobacteria;o__Hydrogenophilales	0.00%	0.00%	0.00%	0.00%	0.00%	0.00%	0.00%	0.00%	0.00%	0.00%	0.00%	0.00%
k__Bacteria;p__Proteobacteria;c__Betaproteobacteria;o__IS-44	0.00%	0.10%	0.10%	0.00%	0.00%	0.10%	0.00%	0.00%	0.00%	0.00%	0.00%	0.10%
k__Bacteria;p__Proteobacteria;c__Betaproteobacteria;o__Procabacteriales	0.00%	0.00%	0.00%	0.00%	0.00%	0.00%	0.00%	0.00%	0.00%	0.10%	0.00%	0.00%
k__Bacteria;p__Proteobacteria;c__Deltaproteobacteria;o__BPC076	0.00%	0.00%	0.00%	0.00%	0.00%	0.00%	0.00%	0.00%	0.00%	0.00%	0.00%	0.00%
k__Bacteria;p__Proteobacteria;c__Deltaproteobacteria;o__Desulfarculales	0.00%	0.00%	0.00%	0.00%	0.00%	0.00%	0.00%	0.00%	0.00%	0.00%	0.00%	0.00%
k__Bacteria;p__Proteobacteria;c__Deltaproteobacteria;o__Desulfobacterales	0.00%	0.00%	0.00%	0.00%	0.00%	0.00%	0.00%	0.00%	0.00%	0.00%	0.00%	0.00%

k__Bacteria;p__Proteobacteria;c__Deltaproteobacteria;o__Desulfovibrionales	0.00%	0.00%	0.00%	0.00%	0.00%	0.00%	0.00%	0.00%	0.00%	0.00%	0.00%	0.00%
k__Bacteria;p__Proteobacteria;c__Deltaproteobacteria;o__FAC87	0.00%	0.00%	0.00%	0.00%	0.00%	0.00%	0.00%	0.00%	0.00%	0.00%	0.00%	0.00%
k__Bacteria;p__Proteobacteria;c__Deltaproteobacteria;o__GMD14H09	0.00%	0.00%	0.00%	0.00%	0.10%	0.00%	0.00%	0.00%	0.00%	0.00%	0.00%	0.10%
k__Bacteria;p__Proteobacteria;c__Deltaproteobacteria;o__GW-28	0.00%	0.00%	0.00%	0.00%	0.00%	0.00%	0.00%	0.00%	0.00%	0.00%	0.00%	0.00%
k__Bacteria;p__Proteobacteria;c__Deltaproteobacteria;o__MIZ46	0.00%	0.00%	0.00%	0.00%	0.00%	0.00%	0.00%	0.00%	0.00%	0.00%	0.00%	0.00%
k__Bacteria;p__Proteobacteria;c__Deltaproteobacteria;o__PB19	0.00%	0.00%	0.00%	0.00%	0.00%	0.00%	0.00%	0.00%	0.00%	0.00%	0.00%	0.00%
k__Bacteria;p__Proteobacteria;c__Deltaproteobacteria;o__Spirobacillales	0.00%	0.00%	0.10%	0.00%	0.00%	0.00%	0.10%	0.10%	0.00%	0.00%	0.10%	0.00%
k__Bacteria;p__Proteobacteria;c__Deltaproteobacteria;o__Sva0853	0.00%	0.00%	0.00%	0.00%	0.00%	0.00%	0.00%	0.00%	0.00%	0.00%	0.00%	0.00%
k__Bacteria;p__Proteobacteria;c__Epsilonproteobacteria;o__Campylobacterales	0.00%	0.00%	0.10%	0.10%	0.00%	0.00%	0.00%	0.00%	0.00%	0.00%	0.00%	0.00%
k__Bacteria;p__Proteobacteria;c__Gammaproteobacteria;Other	0.00%	0.00%	0.00%	0.00%	0.00%	0.00%	0.00%	0.00%	0.00%	0.00%	0.00%	0.00%
k__Bacteria;p__Proteobacteria;c__Gammaproteobacteria;o__34P16	0.00%	0.00%	0.00%	0.00%	0.00%	0.00%	0.00%	0.10%	0.00%	0.00%	0.10%	0.00%
k__Bacteria;p__Proteobacteria;c__Gammaproteobacteria;o__Chromatiales	0.00%	0.00%	0.00%	0.00%	0.00%	0.00%	0.00%	0.00%	0.00%	0.00%	0.00%	0.00%

k__Bacteria;p__Proteobacteria;c__Gammaproteobacteria;o__HOC36	0.00%	0.00%	0.00%	0.00%	0.00%	0.00%	0.00%	0.00%	0.00%	0.00%	0.00%	0.00%
k__Bacteria;p__Proteobacteria;c__Gammaproteobacteria;o__HTCC2188	0.10%	0.00%	0.10%	0.00%	0.00%	0.10%	0.00%	0.00%	0.00%	0.00%	0.00%	0.00%
k__Bacteria;p__Proteobacteria;c__Gammaproteobacteria;o__Methylococcales	0.00%	0.00%	0.00%	0.00%	0.00%	0.00%	0.00%	0.00%	0.00%	0.00%	0.00%	0.00%
k__Bacteria;p__Proteobacteria;c__Gammaproteobacteria;o__Oceanospirillales	0.00%	0.00%	0.00%	0.00%	0.20%	0.00%	0.00%	0.00%	0.00%	0.00%	0.00%	0.00%
k__Bacteria;p__Proteobacteria;c__Gammaproteobacteria;o__Pasteurellales	0.00%	0.00%	0.00%	0.00%	0.00%	0.00%	0.00%	0.00%	0.00%	0.00%	0.00%	0.00%
k__Bacteria;p__Proteobacteria;c__Gammaproteobacteria;o__Vibrionales	0.00%	0.00%	0.00%	0.00%	0.00%	0.00%	0.00%	0.00%	0.00%	0.00%	0.00%	0.00%
k__Bacteria;p__Proteobacteria;c__Gammaproteobacteria;o__[Marinicellales]	0.00%	0.00%	0.00%	0.00%	0.00%	0.00%	0.00%	0.00%	0.00%	0.00%	0.10%	0.10%
k__Bacteria;p__Proteobacteria;c__TA18;o__CV90	0.00%	0.00%	0.00%	0.00%	0.00%	0.00%	0.00%	0.00%	0.00%	0.00%	0.00%	0.00%
k__Bacteria;p__Proteobacteria;c__TA18;o__PHOS-HD29	0.00%	0.00%	0.00%	0.00%	0.00%	0.00%	0.00%	0.00%	0.00%	0.00%	0.00%	0.00%
k__Bacteria;p__SBR1093;c__VHS-B5-50;o__	0.00%	0.00%	0.00%	0.00%	0.00%	0.00%	0.00%	0.00%	0.00%	0.00%	0.00%	0.00%
k__Bacteria;p__SR1;c__;o__	0.00%	0.00%	0.00%	0.00%	0.00%	0.00%	0.00%	0.00%	0.00%	0.00%	0.00%	0.00%
k__Bacteria;p__Spirochaetes;c__[Leptospirae];o__[Leptospirales]	0.00%	0.00%	0.00%	0.00%	0.00%	0.10%	0.00%	0.00%	0.00%	0.00%	0.00%	0.00%
k__Bacteria;p__Synergistetes;c__Synergistia;o__Synergistales	0.00%	0.00%	0.00%	0.00%	0.00%	0.00%	0.00%	0.00%	0.00%	0.00%	0.00%	0.00%

k__Bacteria;p__TM6;c__SBRH58;o__	0.00%	0.00%	0.00%	0.00%	0.00%	0.00%	0.00%	0.00%	0.00%	0.00%	0.00%	0.00%
k__Bacteria;p__TM6;c__SJA-4;o__	0.00%	0.00%	0.00%	0.00%	0.00%	0.00%	0.00%	0.00%	0.00%	0.00%	0.00%	0.00%
k__Bacteria;p__TM6;c__SJA-4;o__S1198	0.00%	0.00%	0.00%	0.00%	0.00%	0.00%	0.00%	0.00%	0.00%	0.00%	0.00%	0.00%
k__Bacteria;p__TM7;Other;Other	0.00%	0.00%	0.00%	0.00%	0.00%	0.00%	0.00%	0.00%	0.00%	0.00%	0.00%	0.00%
k__Bacteria;p__TM7;c__;o__	0.00%	0.10%	0.00%	0.00%	0.00%	0.00%	0.00%	0.00%	0.00%	0.00%	0.00%	0.00%
k__Bacteria;p__TM7;c__MJK10;o__	0.00%	0.00%	0.00%	0.00%	0.00%	0.00%	0.00%	0.00%	0.00%	0.00%	0.00%	0.00%
k__Bacteria;p__TM7;c__SC3;o__	0.00%	0.00%	0.00%	0.00%	0.00%	0.00%	0.00%	0.10%	0.00%	0.00%	0.00%	0.00%
k__Bacteria;p__TM7;c__TM7-3;Other	0.00%	0.00%	0.00%	0.00%	0.00%	0.00%	0.00%	0.00%	0.00%	0.00%	0.00%	0.00%
k__Bacteria;p__TM7;c__TM7-3;o__Blgi18	0.00%	0.00%	0.00%	0.00%	0.00%	0.00%	0.00%	0.00%	0.00%	0.00%	0.00%	0.00%
k__Bacteria;p__TM7;c__TM7-3;o__I025	0.00%	0.00%	0.00%	0.00%	0.00%	0.00%	0.00%	0.00%	0.00%	0.00%	0.00%	0.00%
k__Bacteria;p__Tenericutes;c__Mollicutes;o__Acholeplasmatales	0.00%	0.00%	0.00%	0.00%	0.40%	0.00%	0.00%	0.00%	0.00%	0.00%	0.00%	0.00%
k__Bacteria;p__Tenericutes;c__Mollicutes;o__Anaeroplasmatales	0.00%	0.00%	0.00%	0.00%	0.00%	0.00%	0.00%	0.00%	0.00%	0.00%	0.00%	0.00%
k__Bacteria;p__Tenericutes;c__Mollicutes;o__Mycoplasmatales	0.00%	0.00%	0.00%	0.00%	0.00%	0.00%	0.00%	0.00%	0.00%	0.00%	0.00%	0.00%
k__Bacteria;p__Tenericutes;c__Mollicutes;o__RF39	0.00%	0.00%	0.00%	0.00%	0.00%	0.00%	0.00%	0.00%	0.00%	0.00%	0.00%	0.00%
k__Bacteria;p__Tenericutes;c__RF3;o__ML615J-28	0.00%	0.00%	0.00%	0.00%	0.00%	0.00%	0.20%	0.00%	0.00%	0.00%	0.00%	0.00%
k__Bacteria;p__Verrucomicrobia;Other;Other	0.00%	0.00%	0.00%	0.00%	0.00%	0.00%	0.00%	0.00%	0.00%	0.00%	0.00%	0.00%
k__Bacteria;p__Verrucomicrobia;c__;o__	0.00%	0.00%	0.00%	0.00%	0.00%	0.00%	0.00%	0.00%	0.00%	0.00%	0.00%	0.00%

k__Bacteria;p__Verrucomicrobia; c__Opitutae;o__	0.00%	0.00%	0.00%	0.00%	0.10%	0.00%	0.00%	0.00%	0.00%	0.00%	0.00%	0.00%
k__Bacteria;p__Verrucomicrobia; c__Opitutae;o__[Cerasicoccales]	0.00%	0.00%	0.00%	0.00%	0.00%	0.00%	0.00%	0.00%	0.00%	0.00%	0.00%	0.00%
k__Bacteria;p__Verrucomicrobia; c__Verruco-5;o__LD1-PB3	0.00%	0.00%	0.00%	0.00%	0.00%	0.00%	0.00%	0.00%	0.00%	0.00%	0.00%	0.00%
k__Bacteria;p__Verrucomicrobia; c__Verruco-5;o__WCHB1-41	0.00%	0.00%	0.00%	0.00%	0.00%	0.00%	0.00%	0.00%	0.00%	0.00%	0.00%	0.00%
k__Bacteria;p__Verrucomicrobia; c__[Methylacidiphilae];o__Methylacidiphilales	0.00%	0.00%	0.00%	0.00%	0.00%	0.00%	0.00%	0.00%	0.00%	0.00%	0.00%	0.10%
k__Bacteria;p__Verrucomicrobia; c__[Methylacidiphilae];o__S-BQ2-57	0.00%	0.00%	0.10%	0.00%	0.00%	0.00%	0.00%	0.00%	0.00%	0.00%	0.00%	0.00%
k__Bacteria;p__Verrucomicrobia; c__[Pedosphaerae];Other	0.00%	0.00%	0.00%	0.00%	0.00%	0.00%	0.00%	0.00%	0.00%	0.00%	0.00%	0.00%
k__Bacteria;p__Verrucomicrobia; c__[Pedosphaerae];o__	0.00%	0.00%	0.00%	0.00%	0.30%	0.00%	0.00%	0.00%	0.00%	0.00%	0.00%	0.00%
k__Bacteria;p__WPS-2;c__;o__	0.00%	0.00%	0.00%	0.00%	0.00%	0.00%	0.00%	0.00%	0.00%	0.00%	0.00%	0.00%
k__Bacteria;p__WS2;c__Kazan-3B-09;o__	0.00%	0.00%	0.00%	0.00%	0.00%	0.00%	0.00%	0.00%	0.00%	0.00%	0.00%	0.00%
k__Bacteria;p__WS2;c__SHA-109;o__	0.00%	0.00%	0.00%	0.00%	0.00%	0.00%	0.00%	0.00%	0.00%	0.00%	0.00%	0.00%
k__Bacteria;p__WS3;c__PRR-12;o__	0.00%	0.00%	0.00%	0.00%	0.00%	0.00%	0.00%	0.00%	0.00%	0.00%	0.00%	0.00%
k__Bacteria;p__WS3;c__PRR-12;o__GN03	0.00%	0.00%	0.00%	0.00%	0.00%	0.00%	0.00%	0.00%	0.00%	0.00%	0.00%	0.00%
k__Bacteria;p__WS3;c__PRR-12;o__LD1-PA13	0.10%	0.00%	0.10%	0.00%	0.00%	0.10%	0.00%	0.00%	0.00%	0.00%	0.00%	0.00%
k__Bacteria;p__WS3;c__PRR-12;o__wb1_H11	0.00%	0.00%	0.00%	0.00%	0.00%	0.00%	0.00%	0.00%	0.00%	0.00%	0.00%	0.00%
k__Bacteria;p__WS4;c__;o__	0.00%	0.00%	0.00%	0.00%	0.00%	0.00%	0.00%	0.00%	0.00%	0.00%	0.00%	0.00%

k__Bacteria;p__WS6;c__;o__	0.00%	0.00%	0.00%	0.00%	0.00%	0.00%	0.00%	0.00%	0.00%	0.00%	0.00%	0.00%
k__Bacteria;p__WS6;c__B142;o__	0.00%	0.00%	0.00%	0.00%	0.10%	0.10%	0.00%	0.00%	0.00%	0.00%	0.00%	0.00%
k__Bacteria;p__WS6;c__SC72;o__	0.00%	0.00%	0.00%	0.00%	0.00%	0.00%	0.00%	0.00%	0.00%	0.00%	0.00%	0.00%
k__Bacteria;p__[Caldithrix];c__KSB1;o__Ucn15732	0.00%	0.00%	0.00%	0.00%	0.00%	0.00%	0.00%	0.00%	0.00%	0.00%	0.00%	0.00%
k__Bacteria;p__[Thermi];c__Deinococci;o__Deinococcales	0.00%	0.00%	0.00%	0.00%	0.00%	0.00%	0.10%	0.00%	0.00%	0.00%	0.00%	0.00%

Table S2. Primers used in the study.

For 16S

Oligo Name	Oligo Sequence (5' to 3')	Length of primer (nucleotide unit, n)	Product size (approx.)
Prokaryote V3-Forward	CCTACGGGNBGCASCAG	17	~ 460 bps
Prokaryote V4-Reverse	GACTACNVGGGTATCTAATCC	21	

For ITS2

Oligo Name	Oligo Sequence (5' to 3')	Length of primer (nucleotide unit, n)	Product size (approx.)
ITS2-Forward	GCATCGATGAAGAACGCAGC	20	~ 350 bps
ITS2-Reverse	TCCTCCGCTTATTGATATGC	20	

Table S3. Identified bacterial genera Bacterial genera described as plant-growth promoters were searched on Web of Science (http://www.webofknowledge.com).

Genus	Growth-promoting attribute	Isolated from (organ)	References
Achromobacter	-	-	-
Acidovorax	-	-	-
Acinetobacter	-	-	-
Actinomadura	-	-	-
Actinoplanes	-	-	-
Adhaeribacter	-	-	-
Aeromicrobium	-	-	-
Agrobacterium	-	-	-
Agromyces	-	-	-
Alishewanella	-	-	-
Alkaliphilus	-	-	-
Amaricoccus	-	-	-
Aminobacter	-	-	-
Anaeromyxobacter	-	-	-
Arenimonas	-	-	-
Arthrospira	-	-	-
Azospirillum	-	-	-
Azospirillum	N-fixing	Roots	Molina et al., 2018
Bacillus	IAA production	Roots	Yuan et al., 2018
Balneimonas	-	-	-
Bdellovibrio	-	-	-
Bosea	-	-	-
Bradyrhizobium	N-fixing	Roots	Ulzen et al., 2018
Brevundimonas	-	-	-
Burkholderia	-	-	-
Campylobacter	-	-	-
Candidatus Koribacter	-	-	-
Candidatus Nitrososphaera	-	-	-
Candidatus Solibacter	-	-	-
Candidatus Xiphinematobacter	-	-	-
Caulobacter	-	-	-
Cellulomonas	-	-	-
Cellvibrio	-	-	-
Chelatococcus	-	-	-
Chitinophaga	-	-	-
Chthoniobacter	-	-	-
Citricoccus	-	-	-
Clostridium	-	-	-
Coprococcus	-	-	-
Couchioplanes	-	-	-
Cryocola	-	-	-
Cupriavidus	-	-	-

Dactylosporangium	Antibiotic production	Roots	Fan et al., 2018
Delftia	-	-	-
Demequina	-	-	-
Devosia	-	-	-
Dokdonella	-	-	-
Dyadobacter	N-fixing	Plant	Kumar et al., 2018
Enterobacter	-	-	-
Fimbriimonas	-	-	-
Flavihumibacter	-	-	-
Flavisolibacter	-	-	-
Flavobacterium	-	-	-
Gemmata	-	-	-
Gemmatimonas	-	-	-
Geodermatophilus	-	-	-
Gordonia	-	-	-
Hydrogenophaga	-	-	-
Hylemonella	-	-	-
Hyphomicrobium	-	-	-
Iamia	N-fixing	Roots	Yu et al., 2016
Kaistia	-	-	-
Kaistobacter	-	-	-
Knoellia	-	-	-
Kribbella	-	-	-
Labrys	-	-	-
Lactobacillus	-	-	-
Leptothrix	-	-	-
Limnohabitans	-	-	-
Luteimonas	IAA	Plant	Zhao et al., 2018
Luteolibacter	-	-	-
Mesorhizobium	N-fixing	Roots	Zhang et al., 2018
Methylibium	-	-	-
Methylobacterium	-	-	-
Methylosinus	-	-	-
Methylotenera	-	-	-
Microbacterium	-	-	-
Mycobacterium	-	-	-
Mycoplana	-	-	-
Nitriliruptor	-	-	-
Nitrospira	-	-	-
Nocardia	-	-	-
Nocardioides	-	-	-
Nonomuraea	Antibiotic production	Plant	Zheng et al., 2018
Novosphingobium	-	-	-
Opitutus	-	-	-
Paenibacillus	N-fixing	Plant	Wang et al., 2018
Paracoccus	-	-	-
Pedobacter	-	-	-
Pedomicrobium	-	-	-
Phaeospirillum	-	-	-
Phenylobacterium	-	-	-
Phycicoccus	-	-	-

Phyllobacterium	N-fixing	Roots	Safronova et al., 2018
Phytohabitans	Antibiotic production	Plant	Qin et al., 2015
Pilimelia	-	-	-
Pimelobacter	-	-	-
Pirellula	-	-	-
Planctomyces	-	-	-
Pleomorphomonas	-	-	-
Polaromonas	-	-	-
Pontibacter	-	-	-
Promicromonospora	-	-	-
Pseudomonas	IAA	Roots	Kavino et al., 2018
Pseudonocardia	-	-	-
Pseudoxanthomonas	-	-	-
Ramlibacter	-	-	-
Rhizobium	N-fixing	Roots	Yuan et al., 2018
Rhodobacter	-	-	-
Rhodococcus	-	-	-
Rhodoplanes	-	-	-
Rubrobacter	-	-	-
Shinella	-	-	-
Skermanella	-	-	-
Solirubrobacter	-	-	-
Sphingobium	-	-	-
Sphingomonas	-	-	-
Sphingopyxis	-	-	-
Sporomusa	-	-	-
Sporosarcina	-	-	-
Steroidobacter	-	-	-
Streptacidiphilus	-	-	-
Streptomyces	-	-	-
Streptosporangium	-	-	-
Terracoccus	-	-	-
Terrimonas	-	-	-
Thermomonas	-	-	-
Variovorax	IAA	Plant	Zhou et al., 2017
Virgisporangium	-	-	-

Table S4. Identified fungal genera. Fungal genera described as plant-growth promoters were searched on Web of Science (http://www.webofknowledge.com).

Genus	Growth-promoting attribute	Isolated from (organ)	References
Acremonium	Biocontrol	Plant	Nandhini et al., 2018
Acrostalagmus	-	-	-
Agaricus	-	-	-
Alternaria	-	-	-
Aspergillus	Antioxidant	Roots	Giorni et al., 2007
Auricularia	-	-	-
Botryosphaeria	-	-	-
Ceratocystis	-	-	-
Clitopilus	-	-	-
Cordyceps	-	-	-
Cosmospora	-	-	-
Cylindrocarpon	-	-	-
Cyphellophora	-	-	-
Echinoderma	-	-	-
Emericella	-	-	-
Eurotium	-	-	-
Exophiala	-	-	-
Fusarium	-	-	-
Gibberella	-	-	-
Gymnopilus	-	-	-
Haematonectria	-	-	-
Humicola	-	-	-
Leucoagaricus	-	-	-
Marasmius	-	-	-
Mortierella	-	-	-
Myrothecium	-	-	-
Neosartorya	-	-	-
Penicillium	-	-	-
Phialophora	-	-	-
Phoma	-	-	-
Plectosphaerella	-	-	-
Schizophyllum	-	-	-
Septoria	-	-	-
Setosphaeria	-	-	-
Termitomyces	Bioactive compounds	Plant	Hsieh and Ju 2018
Trichoderma	Biocontrol	Roots	Sangeetha et al., 2009
Xeromyces	-	-	-
	-	-	-
